# Polygoni Multiflori Radix interferes with bile acid metabolism homeostasis by inhibiting *Fxr* transcription, leading to cholestasis

**DOI:** 10.3389/fphar.2023.1099935

**Published:** 2023-03-06

**Authors:** Yihang Dai, Zhixin Jia, Cong Fang, Meixia Zhu, Xiaoning Yan, Yinhuan Zhang, Hao Wu, Menghan Feng, Lirong Liu, Beibei Huang, Yueting Li, Jie Liu, Hongbin Xiao

**Affiliations:** ^1^ School of Chinese Materia Medica, Beijing University of Chinese Medicine, Beijing, China; ^2^ Research Center of Chinese Medicine Analysis and Transformation, Beijing University of Chinese Medicine, Beijing, China; ^3^ Beijing Academy of Traditional Chinese Medicine, Beijing, China

**Keywords:** Radix Polygoni Multiflori, cholestasis, bile acid metabolism, farnesoid X receptor, cholesterol-7α-hydroxylase, bile salt output pump

## Abstract

**Objective:** To explore the possible mechanisms of cholestasis induced by Polygoni Multiflori Radix (PM).

**Methods:** Low and high doses of water extract of PM were given to mice by gavage for 8 weeks. The serum biochemical indexes of aspartate aminotransferase (AST), alanine aminotransferase (ALT), glutamyltransferase (GGT) alkaline phosphatase (ALP) and so on were detected in the second, fourth, sixth, and eighth weeks after administration. At the end of the eighth week of administration, the bile acid metabolic profiles of liver and bile were screened by high-performance liquid chromatography tandem triple quadrupole mass spectrometry (HPLC-QQQ-MS/MS). Liver pathological changes were observed by hematoxylin and eosin staining. Real-time quantitative polymerase chain reaction (RT-qPCR) was used to detect the mRNA transcription of the target genes and Western blotting (WB) was used to the detect target protein expression.

**Results:** Biochemical tests results showed the values of ALP and GGT were two and three times greater than the normal values respectively, and the value of R was less than 2. Histopathology also showed that PM caused lymphocyte infiltration, a small amount of hepatocyte necrosis and nuclear fragmentation in mouse liver. The proliferation of bile duct epithelial cells was observed in the high group. These results indicated that PM may lead to cholestatic liver injury. HPLC-QQQ-MS/MS analysis with the multivariate statistical analysis revealed significant alterations of individual bile acids in liver and gallbladder as compared to those of the control group. RT-qPCR showed that the transcription of *Fxr*, *Shp*, *Bsep*, *Bacs*, *Mdr2*, and *Ugt1a1* were downregulated and that of *Cyp7a1*, *Mrp3*, and *Cyp3a11* was significantly upregulated in the treatment group. WB demonstrated that PM also markedly downregulated the protein expression of FXR, BSEP, and MDR2, and upregulated CYP7A1.

**Conclusion:** PM inhibited the expression of FXR, which reduced the expression of MDR2 and BSEP, leading to the obstruction of bile acids outflow, and increased the expression of CYP7A1, resulting in an increase of intrahepatic bile acid synthesis, which can lead to cholestasis.

## 1 Introduction

Polygoni Multiflori Radix (PM) is the dry root tuber of *Polygonum multiflorum* Thunb., which was first recorded in Kaibao Bencao. It has a long history of used in the clinical practice of traditional Chinese medicine. According to the Chinese Pharmacopoeia (2020 edition), PM help in detoxification, carbuncle elimination, malaria prevention and bowel relaxation, and its processed products (Polygoni Multiflori Radix Praeparata) nourish liver and kidneys, and aid in essence and blood benefiting and hair blackening. PM and its processed products are not only used in traditional Chinese medicine, but also in some healthcare products. There were 61 drug preparations containing PM or its processed products, as recorded in the Chinese Pharmacopoeia (2020 edition). These preparations are mainly used for cardiovascular and cerebrovascular diseases, tumors, diabetes, and neurodegenerative diseases (Xue et al., 2020).

However, in recent years, with increasing reports about liver injury caused by PM (Jung et al., 2011; Chen, 2013; Dong et al., 2014; Lei et al., 2015), which have attracted worldwide attention toward PM safety as a commonly used traditional Chinese medicine (Ma et al., 2020). The mechanism underlying the induction of liver damage due to the complex manifestations of liver injury induced by PM remains not completely clear. Three types (liver cell injury type, cholestasis type, and mixed type) of injuries have been observed in clinics. Moreover, Acute and cholestatic hepatitis are the main pathological types, and chronic hepatitis is rare (Tong, 2015; Branch of Chinese Patent Medicine et al., 2020). It has been reported that the clinical manifestations of liver injury induced by PM are jaundice and cholestasis in different degrees, suggesting that its hepatotoxicity may be related to cholestasis (Lin et al., 2015).

At present, research on liver injury caused by PM mostly focuses on the type of liver cell injury, and researchers are concerned about the possible direct cytotoxicity of anthraquinones (Li et al., 2012; Liu et al., 2015; Lv et al., 2015). Although these studies show that PM may cause direct hepatocyte injury to some extent, animal and hepatocyte models based on inherent toxicity cannot accurately simulate and explain the clinical characteristics. This means that direct hepatocyte injury may only partially explain the liver injury of PM. While research on cholestasis caused by PM is rare. Therefore, there is a need to study the cholestasis induced by PM.

Cholestasis is a syndrome characterized by jaundice caused by the obstruction of bile flow due to various causes, which leads to the failure of bile to enter the duodenum normally. Bile acid is the main substance in bile. Its function and homeostasis are associated with liver injury (Chiang, 2017). Cholestasis is the main form of drug-induced liver injury, and damage to bile acid homeostasis is the main cause of drug-induced cholestasis (Hoofnagle and Bjornsson, 2019).

Early studies have shown that the process of hepatocyte injury induced by hydrophobic bile acids involves the participation of the mitochondrial pathway, endoplasmic reticulum stress and death receptor pathway, which may be related to apoptosis and necrosis of hepatocytes (Yerushalmi et al., 2001; Allen et al., 2011). Recent studies have found that conjugated bile acids are significantly correlated with cholestatic liver fibrosis and liver cancer. For example, glycine conjugates of free bile acids may induce the production of reactive oxygen species, eventually leading to hepatocyte apoptosis (Deferm et al., 2019). Conjugated bile acids and estrogen can activate sphingosine 1-phosphate receptor 2, stimulate the ERK signaling pathway, promote the development of liver fibrosis and cause cholestatic liver injury (Li et al., 2017). More seriously, bile acid accumulation can damage bile duct cells and inhibit their differentiation and proliferation of bile duct cells (He et al., 2019).

Bile acid homeostasis is critically regulated by the farnesoid X receptor (FXR) which is the key steps in the production and enterohepatic circulation of bile acids (Zhu et al., 2016). FXR mediates its effects on bile acid metabolism *via* direct induction of target genes and indirect repression *via* the induction of a small heterodimer partner (SHP) (Calkin and Tontonoz, 2012). The activation of FXR can inhibit bile acid synthase, such as CYP7A1 and CYP8B1, through the small heterodimeric anthraquinone (SHP) or fibroblast growth factor 15/19 pathway and intrahepatic flow transporter (NTCP and OATP) (Shin and Wang, 2019). FXR can also upregulate the liver efflux transporters BSEP, MRP2 and MDR2, as well as the liver detoxification enzymes UDP glucuronosyltransferase (UGT1A1 and UGT2B5), sulfur transferase (SULT2A1), and liver drug metabolism enzymes (CYP3A11 and CYP2B10) to reduce intrahepatic bile acid overload and cholestatic liver injury. In contrast, research has confirmed that after inhibition of FXR or FXR gene knockout, bile acid synthase and uptake transporter increase, while bile acid efflux transporter and liver detoxification enzymes decrease, resulting in bile acid overload and reduced bile acid detoxification ability, leading to cholestatic liver injury (Cariello et al., 2018).

Therefore, in view of the unclear mechanism of cholestatic liver injury by PM, we propose that cholestasis may be an important mechanism. We intended to verify this hypothesis from the perspective of bile acid targeted metabolomics, gene transcription, and protein expression levels.

## 2 Materials and methods

### 2.1 Plant material and chemical reagents

PM was purchased from Chinese Herbal Medicine Co., Ltd. of Guangdong province and authenticated by Prof. Xueyong Wang (School of Chinese Materia Medica, Beijing University of Chinese Medicine, Beijing, 102488, China). The voucher specimens (numbered BUCM-HSW-202010) are displayed in the Center for Analysis and Transformation of Chinese Medicine of Beijing University of Chinese Medicine.

Nineteen target bile acids (BAs) and internal standard (IS) are as follows: deoxycholic acid (DCA-d4, IS, batch No. J29GS153010), taurochenodeoxycholic acid (TCDCA, batch No.Y09S8K43540), glycocholic acid (GCA, batch No.Y08A9E57765), lithocholic acid (LCA, batch No.B24S10J98701), glycoursodeoxycholic acid (GUDCA, batch No. B03D9K76539), ursodeoxycholic acid (UDCA,batch No.Y16J7C17898), taurocholic acid sodium salt (TCA, batch No. M02J9E52008), glycochenodeoxycholic acid (GCDCA, batch No.Y29M9K57235), sodium taurolithocholate (TLCA, batch No.Y06N9K74072), sodiumglycodeoxycholate (GDCA, batch No.Y27M9E56860), glycylhyodeoxycholic acid (GHCA, batch No. Y23N9K75826),hyodeoxycholic acid (HCA, batch No. S27M8I36899) and physcion (batch No. M15GB141118) were purchased from Shanghai yuanye BioTechnology Co., Ltd. (Shanghai, China). Sodium taurodeoxycholate (TDCA, batch No. R6500005) was purchased from ANPEL Laboratory Technolgies Inc. (shanghai, China). Dehydrocholic acid (DHCA, batch No. C11067669) and β-muricholic acid (β-MCA, batch No.C12860190) were purchased from Shanghai Macklin Biochemical Co., Ltd. (Shanghai, China). Deoxycholic acid (DCA, batch No. RFS-Q01311804026), chenodeoxycholic acid (CDCA, batch No. RFS-E01611804026), cholicacid (CA, batch No. RFS-D04511812016), tauroursodeoxycholic acid (TUDCA, batch No. RFS-N01411804020), taurohyodeoxycholic acid (THCA, batch No. RFS-N04102008020), emodin (batch No. RFS-D02901905016), and stilbene glycosides (batch No. RFS-E02202103029) were purchased from Chengdu Herbpurify Co., Ltd. (Chengdu, China). Methanol (LC-MS grade) was bought from Merck (Darmstadt, Germany). Ammonium acetate was purchased from Aladdin (Shanghai, China). Deionized water purified from Millipore was used for all dilutions (Milli-Q, Millipore Bedford, MA, United States). ALT, AST, ALP, TBIL (total bilirubin), TBA (total bile acid) and DBIL (direct bilirubin) (batch No. AUZ3777) were purchased from Beckman Coulter Experimental System (Suzhou) Co., Ltd. GGT (batch No. 20200913) was purchased from Nanjing Jian Cheng Bioengineering Institute (Nanjing, China).

The primers were synthesized by Sangon Biotech (Shanghai, China) Co., Ltd. The primer sequences used in this study are listed in Table 2. Invitrogen trizol was purchased from Thermo Fisher Scientific Inc. (Shanghai, China). HiScript III RT SuperMix for qPCR (+gDNA wiper) (R323-01) and ChamQ Universal SYBR qPCR Master Mix (Q711) were purchased from Vazyme (Nanjing, China).

Prestained color protein marker (15-120kD, 6.5-270kD) were purchased from BEIJING LABLEAD BIOTECHNOLOGY CO., LTD (Beijing, China). Native PAGE electrophoresis buffer with tris-gly, bicinchoninic acid protein (BCA) assay kits, RIPA lysis buffer, western rapid transfer buffer (powder) and TBST were purchased from Beyotime Biotechnology (Shanghai, China). Mouse anti-GAPDH (TA-08) and peroxidase-conjugated goat anti-mouse LgG (H+L) (ZB-2305) were purchased from Beijing Zhongshan Goldenbridge Biotechnology Co., Ltd. (Beijing, China). SDS-PAGE gel parparation kit and ECL Western blot kit were purchased from Beijing ComWin BiotechCo., Ltd. (Beijing, China). Anti-P4507A1 (Ab-DF2612) was purchased from Affinity Bioscience (Shanghai, China). Anti-NR1H4 (25055-1-AP) was purchased from Proteintech Group Inc. (Wuhan, China). Anti-MDR2 (sc-58221) and anti-BSEP (sc-74500) were purchased from Santa Cruz Biotechnology (Shanghai) Co., Ltd. (Shanghai, China).

### 2.2 PM extract preparation, qualitative analysis of anthraquinones and stilbene glycosides in the extract by ultrahigh performance liquid chromatography tandem quadrupole time-of-flight mass spectrometry (UHPLC-Q-TOF-MS/MS) and the quantative analysis, fingerprint by high performance liquid chromatography-ultraviolet detector (HPLC-UV)

PM was sliced into small pieces, 600 g of which was mixed with distilled water at a solid-liquid ratio of 1:10, refluxed for 1.5 h and filtered. The extraction process was repeated twice. The combined filtrates were concentrated using a rotary concentrator to remove water, and the obtained concentrate was dried in a vacuum dryer to obtain the extract. The extraction rate of PM extract is 27.24%–31.87%. Approximately 1 g of the extract was carefully weighed, dissolved in 70% (v/v) methanol and diluted to 100 mL with methanol using a volumetric flask. All solutions were filtered through a 0.22 µm filter membrane for the UHPLC-Q-TOF-MS/MS analysis. The sample detection was performed by an Agilent 1,290/6,550 iFunnel Q-TOF MS system with a negative ionization mode. UHPLC analysis was performed on a waters ACQUITY UPLC HSST-3 C18 analytical column (2.1 mm × 100 mm, 1.8 µm, kept at 37°C). Mobile phases consisted of water with 0.1% formic acid (solvent A) and acetonitrile with 0.1% formic acid (solvent B) using the following gradient elution: 0–2 min, 2% B–8% B; 2–10 min, 8% B–25% B; 10–15 min, 25% B–35% B; 15–20 min, 35% B–85% B; 20–27 min, 85% B–90% B; 27–30 min, 90% B–100% B. The flow rate was 0.3 mL/min, the injection volume was 2 μL, and column oven was maintained at 35°C. The Dual AJS ESI source parameters were as follows: gas temperature, 250°C; gas flow, 9 L/min; nebulizer pressure, 35 psi; sheath gas temperature, 300°C; sheath gas flow, 11 L/min; capillary voltage, 3,500 V(−)/4,000 V(+); nozzle voltage, 1,000 V; fragment voltage, 380 V; MS range, 100–1,000 m/z. The sample collision energy was set at 10, 20, and 40 V. The mass spectral data were processed by Agilent Mass Hunter Qualitative Analysis B.07.00 software (version B.07.00, Agilent Technologies, United States). We referred to the liquid chromatography detection method established by our research group in the early stage to detect the content of emodin, stilbene glycosides and physcion in the extract of PM (Zhong et al., 2016). These three compounds are the index components of PM marked in the Chinese Pharmacopoeia. Based on this method, we established the fingerprint of extracts under three ultraviolet detection wavelengths.

### 2.3 Animal experiment

ICR mice of SPF grade in male about 8 weeks old with the weight between 30 g and 35 g [certificate number: SCXK (Jing) 2019-0009] were purchased from Beijing Vital River Laboratory Animal Technology Co., Ltd. (Beijing, China). All mice were housed in an SPF grade animal room at a temperature of 25°C ± 2°C and relative humidity of 60%. All the mice were fed SPF-grade food and sterile distilled water. The animal study protocols were approved by the Animal Ethical and Welfare Committee of the Beijing University of Chinese Medicine (approval number: BUCM-4-2020082503-3150).

An 8-week oral administration experiment was conducted to study the cholestatic hepatotoxicity induced by PM extract (PM-Ex) in mice. The body weights of mice were measured after a 7-day quarantine period, and the animals were randomly divided into three groups (12 mice per group). The groups were denoted as the vehicle group, low-dose of PM-Ex group (PM-L), and high-dose of PM-Ex group (PM-H). In the PM-L group, 12 mice were orally administered 2.5 g/kg PM-Ex once per day; in the PM-H group, 12 mice were orally administered 5 g/kg PM-Ex; and in the vehicle group, 12 mice were orally administrated an equivalent volume of distilled water.

### 2.4 Serum biochemistry analysis

At the end of the second, fourth, sixth, and eighth week after administration, the mice were fasted for 12 h, and 100 µL of blood was collected from the inner canthus after anesthesia. After incubating at 26°C for an hour, the blood was centrifuged at 3500 rpm for 15 min at 4°C, and the supernatant was collected to obtain serum samples for biochemical analysis. The value of GGT was detected by a TECAN Nano Quant microplate analyzer (TECAN, Tecan Trading AG, Switzerland). The values of ALT, AST, ALP, TBA, TBIL, and DBIL were quantified with a BECKMAN COULTER AH480 biochemical autoanalyser (Beckman Coulter, Kraemer Boulevard Brea, United States).

### 2.5 Histopathology analysis

At the end of 8 weeks of treatment, we collected the livers of mice that were sacrificed by cervical dislocation. Partial liver tissues were preserved for histological analysis. These part of liver tissue were fixed in 4% paraformaldehyde, stained with hematoxylin and eosin (H&E), and then examined by an Olympus microscope. Pathologists were blinded to the H&E stained images. The incidence of hepatic lesions was determined by histological analysis of the same portion of the liver in each animal.

### 2.6 Metabolomics of bile acids based on HPLC-QQQ-MS/MS

#### 2.6.1 Sample preparation

At the end of 8 weeks of treatment, each animal was fasted for 12 h. Blood was collected from the mice after anesthesia. The Partial liver and gallbladder were collected and stored at −80°C before HPLC-QQQ-MS/MS analysis. The frozen gallbladder contents (10 µL) were mixed with 90 µL cold normal saline for dilution, 500 µL cold methanol and 10 µL IS were added, and the frozen liver tissue (100 mg) was added to 500 µL physiological saline to prepare the homogenate, which was added to 5 times amount of cold methanol with 10 µL IS. The mixture was vortexed for 10 min and centrifuged at 12,000 r/min for 10 min at 4°C. The solvent was evaporated in a rotary vacuum concentrator. Methanol solution (50 µL) was added for re-dissolution, centrifuged again at the same condition, and the supernatant was analyzed using HPLC-QQQ-MS/MS.

#### 2.6.2 HPLC-QQQ-MS/MS profiling and MS conditions for bile acids analysis

**FIGURE 1:**
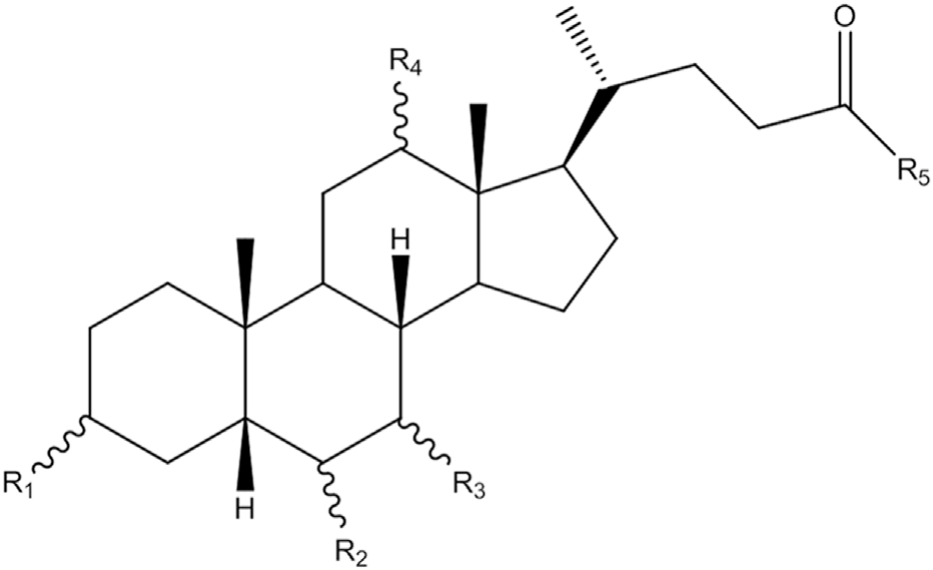
The structure of bile acids.

MS analysis of bile acids was performed on an Agilent 1,260 series HPLC system (Agilent Technologies, Santa Clara, CA, United States) equipped with a quaternary pump, degasser, auto sampler and thermostatically controlled column compartment. Chromatographic separation was performed on an Agilent Poroshell 120 EC-C18 (3.0 mm × 150 mm, 2.7 micron) at 37°C. The structures of bile acids are shown in Figure 1 and Table 1. The mobile phase consisted of 5 mmol/L ammonium acetate containing 0.1% formic acid (solvent A) and methanol (solvent B). A gradient elution program was used as follows: linear gradient from 0 to 2 min, 25% B–46% B; 2–27 min, 46% B–70% B; 27–35 min, 70% B−95% B; 35–38 min, 95% B–98% B. The flow rate was 0.3 mL/min and the injected sample volume was 3 µL. Detection was achieved using an Agilent triple quadrupole 6,470 mass spectrometer (MS) with an electrospray ionization source. The main working parameters for MS were set as follows: drying gas (N_2_) flow rate, 10 L/min; drying gas temperature, 300°C; nebulizing gas (N_2_) pressure, 45 psi; capillary voltage, 3,000 V; quadrupole temperature, 300°C. Fragment ion spectra were recorded by negative electrospray ionization in the multiple reaction monitoring mode (MRM) and MS parameters, as shown in Table 2.

**TABLE 1:** The substituent group of different bile acids.

Bile acids	R_1_	R_2_	R_3_	R_4_	R_5_
CA	α-OH	H	α-OH	α-OH	OH
CDCA	α-OH	H	α-OH	H	OH
DCA	α-OH	H	H	OH	OH
DCA-d_4_(IS)	α-OH	H	H	OH	OH
DHCA	=O	H	=O	=O	OH
HCA	α-OH	α-OH	α-OH	H	OH
LCA	α-OH	H	H	H	OH
UDCA	α-OH	H	β-OH	H	OH
β-MCA	α-OH	β-OH	β-OH	H	OH
TCA	α-OH	H	α-OH	OH	NH(CH_2_)_2_SO_3_H
TCDCA	α-OH	H	α-OH	H	NH(CH_2_)_2_SO_3_H
TDCA	α-OH	H	H	OH	NH(CH_2_)_2_SO_3_H
THCA	α-OH	α-OH	α-OH	α-OH	NH(CH_2_)_2_SO_3_H
TLCA	α-OH	H	H	H	NH(CH_2_)_2_SO_3_H
TUDCA	α-OH	H	β-OH	H	NH(CH_2_)_2_SO_3_H
T-β-MCA	α-OH	β-OH	β-OH	H	NH(CH_2_)_2_SO_3_H
GCA	α-OH	H	α-OH	OH	NHCH_2_COOH
GCDCA	α-OH	H	α-OH	H	NHCH_2_COOH
GDCA	α-OH	H	H	OH	NHCH_2_COOH
GHCA	α-OH	α-OH	α-OH	α-OH	NHCH_2_COOH
GUDCA	α-OH	H	β-OH	H	NHCH_2_COOH

**TABLE 2:** MRM transitions and MS parameters in negative ion mode for all bile acids.

Bile acids	Ion pair	Fragment (V)	CE (eV)
CA	407.3 -> 343.3	190	15
CDCA	437.3 -> 391.3	165	25
DCA	391.3 -> 345.2	175	50
DCA-d_4_(IS)	395.3 -> 395.3	180	15
DHCA	401.1 -> 249.2	210	75
HCA	437.3 -> 391.3	80	19
LCA	375.3 -> 375.2	120	10
UDCA	437.3 -> 391.3	165	25
β-MCA	407.3 -> 407.3	170	30
TCA	514.3 -> 79.7	295	90
TCDCA	498.3 -> 79.8	295	90
TDCA	498.3 -> 79.8	295	85
THCA	498.3 -> 79.8	290	90
TLCA	483.3 -> 79.9	285	85
TUDCA	498.3 -> 79.8	280	86
T-β-MCA	514.3 -> 80.0	205	45
GCA	464.3 -> 73.7	170	55
GCDCA	448.3 -> 74.0	145	35
GDCA	448.3 -> 74.0	190	60
GHCA	448.3 -> 74.0	170	45
GUDCA	448.3 -> 74.0	145	35

#### 2.6.3 HPLC-QQQ-MS/MS method validation

Bile acid-free samples were prepared using a modified method described previously (Yang et al., 2008; Yang et al., 2017). Bile acid-free bile matrix preparation: blank mice bile (50 µL) was mixed with physiological saline (5 mL) and biologically activated charcoal (0.75 g). The mixture was vortexed for 1 h, centrifuged at 1,000 g for 20 min, and the supernatant was collected (this procedure was repeated twice). The supernatant was used as the bile acid-free bile matrix solution. To prepare the bile acid-free liver matrix, blank liver samples (1 g) were homogenized in 5 mL of physiological saline. The mixture was centrifuged at 4,000 rpm for 10 min, and the supernatant was collected, mixed with 3 g of activated charcoal, and shaken for 1 h. The mixture was centrifuged (1,000 × g) for 20 min and the supernatant was collected (this procedure was repeated twice). The supernatant was used as bile acid-free liver matrix solution.

Preparation of calibration standards and quality control (QC) samples: stock solutions were prepared by dissolving accurately weighed 19 BAs in methanol. The stock solutions were then diluted into a series of standard solutions of appropriate concentrations. Each diluted solution (10 µL) was spiked into 90 µL bile acid-free matrix solution for calibration analysis. QC samples with low, medium, and high concentrations were prepared in the same manner. IS solution was prepared in methanol at a concentration of 10 μg/mL.

According to the “Guidance for Industry-Bioanalytical Method Validation” recommended by the USFDA (2001), HPLC-QQQ-MS/MS method validation was performed to evaluate the selectivity, linearity, sensitivity, precision, accuracy, recovery and matrix effect. The selectivity was tested by analyzing the chromatograms of bile free bile and liver, bile and liver mixed with IS, and bile and liver samples after oral PM. The linearity of each calibration curve was determined by plotting the peak area ratio (y) of the analytes to IS versus the nominal concentration (x) of the analysis using weighted least square linear regression. The sensitivity of the method was determined using the lower limit of quantification (LLOQ). The LLOQ of the assay was defined as the lowest concentration on the calibration curve with an acceptable accuracy within ±20% and precision below 20%. Precision and accuracy were assessed by analyzing QC samples. Intraday precision and accuracy were evaluated by six replicates of QC samples on the same day. The precision was expressed as the relative standard deviation, and accuracy was expressed as the relative error. The extraction recoveries of the analysis at three QC levels were determined by comparing the peak areas obtained from the extracted QC samples with those obtained from pure reference standards spiked in post-extracted BAs-free mice bile and liver at the same concentration. The matrix effect was determined by comparing the peak areas obtained from the samples in which the extracted matrix was mixed with the standard solution and the peak area obtained from the pure reference standard solution of the same concentration. The results of the methodological validation are provided in the Supplementary Material.

#### 2.6.4 HPLC-QQQ-MS/MS data acquisition and statistical analysis

HPLC-QQQ-MS/MS raw data were analyzed using Mass Hunter Workstation 10.00 (Agilent Technologies). Statistically significant differences in mean values were tested using Student’s *t*-test. Principal components analysis (PCA) and orthogonal partial least (OPLS-DA) were performed using the SIMCA-P software package (version 14.1, Umetrics AB, Umea, Sweden).

### 2.7 Real-time quantitative polymerase chain reaction analysis

Real-time quantitative polymerase chain reaction (RT-qPCR) analysis provides mechanistic insight into liver injury. Firstly, we used the online primer retrieval system of Stanford University to design and screen primers (https://pga.mgh.harvard.edu/primerbank). All primer sequences were listed in Table 3. Total RNA was isolated from liver tissue by using trizol reagent. Then the total RNA concentration and purity were measured using nano-100 (zero with DEPC water for dissolving RNA before measurement). Subsequently, first-strand cDNA synthesis was performed using the HiScript III RT Super Mix. RT-qPCR was performed using ChamQ Universal SYBR qPCR Master Mix. The RT-qPCR procedure was as follows: 40 cycles of pre-denaturation for 30 s at 95°C, 95°C for 10 s, and 60°C for 30 s. *Gapdh* was used as an internal control to measure the transcription levels of the target genes. All assays were performed in duplicates. The relative gene transcription was calculated using the 2^−ΔΔCT^ method. Data are presented as fold-differences relative to the control group.

**TABLE 3:** Primer sequence.

	Gene	Forward primer	Reverse primer
Transcription factor	*Fxr*	GGC​AGA​ATC​TGG​ATT​TGG​AAT​CG	GTT​GTC​CAA​AGG​AGG​TTC​ACC
	*Shp*	TGG​GTC​CCA​AGG​AGT​ATG​C	GCT​CCA​AGA​CTT​CAC​ACA​GTG
Synthesis	*Cyp7a1*	GCT​GTG​GTA​GTG​AGC​TGT​TG	GTT​GTC​CAA​AGG​AGG​TTC​ACC
	*Cyp27a1*	CCA​GGC​ACA​GGA​GAG​TAC​G	GGG​CAA​GTG​CAG​CAC​ATA​G
	*Cyp8b1*	CTA​GGG​CCT​AAA​GGT​TCG​AGT	GTA​GCC​GAA​TAA​GCT​CAG​GAA​G
	*Hsd3b7*	AGG​CCA​GTC​CAA​AGA​CCA​TC	TGC​TCG​TGT​AGA​CCA​GGT​ACT
Metabolize	*Cyp3a11*	CTT​GGT​GCT​CCT​CTA​CCG​ATA​TG	TGG​GTC​TGT​GAC​AGC​AAG​GA
	*Ugt1a1*	GCT​TCT​TCC​GTA​CCT​TCT​GTT​G	GCT​GCT​GAA​TAA​CTC​CAA​GCA​T
	*Baat*	GTG​CTG​GTG​GAT​TGA​TGG​AGT	CCG​AGG​ACC​TTA​GGA​TGT​CTC
	*Bacs*	CTA​CGC​TGG​CTG​CAT​ATA​GAT​G	CCA​CAA​AGG​TCT​CTG​GAG​GAT
Transporter	*Mrp2*	GTG​TGG​ATT​CCC​TTG​GGC​TTT	CAC​AAC​GAA​CAC​CTG​CTT​GG
	*Mdr2*	GAC​ACT​GTT​CCG​ATA​CTC​TGA​CT	ACC​TGA​TCC​ATG​AGC​TAT​GGC
	*Mrp3*	GTC​CCC​TGC​ATC​TAC​CTG​TG	GCC​GTC​TTG​AGC​CTG​GAT​AA
	*Bsep*	TCT​GAC​TCA​GTG​ATT​CTT​CGC​A	CCC​ATA​AAC​ATC​AGC​CAG​TTG​T
	*Ntcp*	CAA​ACC​TCA​GAA​GGA​CCA​AAC​A	GTA​GGA​GGA​TTA​TTC​CCG​TTG​TG
Reference	*Gapdh*	TGG​CCT​TCC​GTG​TTC​CTA​C	GAG​TTG​CTG​TTG​AAG​TCG​CA

### 2.8 Western blot analysis

Mouse liver tissues were homogenized in 1X RIPA lysis buffer containing protease inhibitors to extract total proteins. The protein concentrations were determined using a BCA assay kit to ensure equivalent total protein. The target proteins were separated by 10% or 8% SDS-PAGE and wet-transferred to a polyvinylidene fluoride membrane (Merck Millipore, Darmstadt, Germany). The bands were combined with GAPDH, FXR, BSEP, CYP7A1, and MDR2 primary anti-bodies, incubated overnight at 4°C, and probed with secondary isotope specific antibodies tagged with horseradish peroxidase for 1 h at room temperature. Protein bands were visualized by an enhanced chemiluminescence imaging system using ECL Plus detection reagents (ChemiScope Mini, Shanghai, China) and quantified using ImageJ software.

### 2.9 Statistical analysis

All data were expressed as mean ± standard error of the mean (SEM). Paired Student’s *t*-test and analysis of variance (ANOVA) were performed to evaluate the differences between groups. Statistical analysis was performed using Statistical Product and Service Solutions (SPSS) software (SPSSversion22, Chicago, IL, United States). * indicates a significant difference compared to the control group (*p < 0.05, **p < 0.01). SIMCA-P (SIMCA-P 14.1, Malmo, Sweden) software is used for multivariate statistical analysis of data.

## 3 Results

### 3.1 The qualitative analysis of anthraquinones and stilbene glycosides in the extract using UHPLC-Q-TOF-MS/MS, and the quantative analysis, fingerprint by HPLC-UV

To conduct quality control analysis on the test sample of extract, we have selectively identified 21 stilbene glycosides and anthraquinones from PM on the basis of the accurate mass measurements, fragmentation behavior, reference standards and related literature consulting. These detected components were consistent with the suspected liver injury components of PM reported in the literature (Liu et al., 2018). The contents of emodin, stilbene glycoside and physcion in PM extract were detected at 372.8 μg/g, 14,988.3 μg/g and 38.8 μg/g, respectively. We detected fingerprint patterns of extracts at three different UV detection wavelengths. The details are provided in the Supplementary Material.

### 3.2 Effect of PM on serum biochemical indexes

**FIGURE 2:**
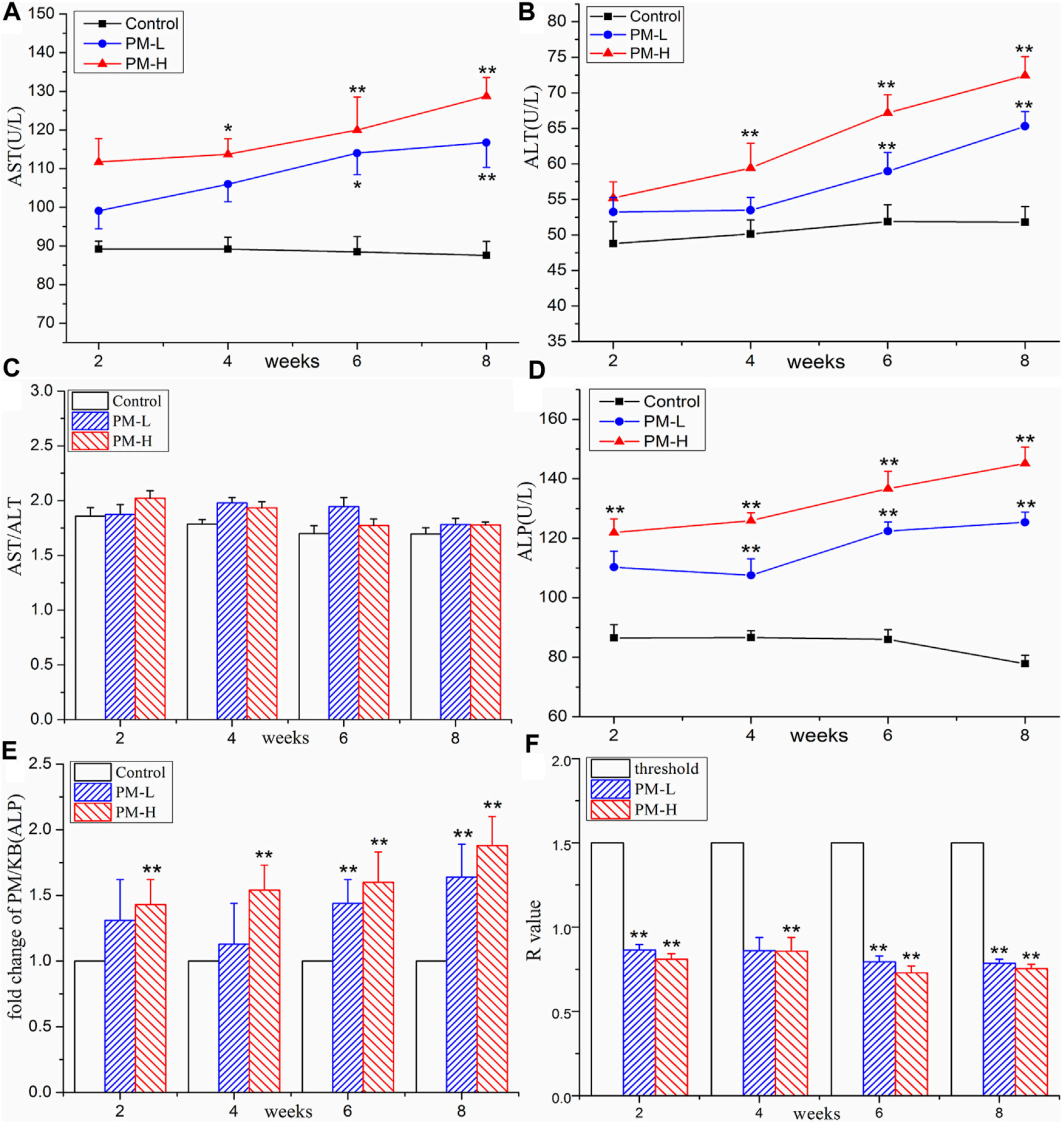
Contents of AST **(A)**, ALT **(B)**, AST/ALT ratio **(C)**, ALP **(D)**, fold change of ALP **(E)** and R values **(F)** in Control, PM-L and PM-H group at the end of second, fourth, sixth and 8 week after administration. (mean ± SEM, *n* = 8, *p < 0.05, **p < 0.01).

**FIGURE 3:**
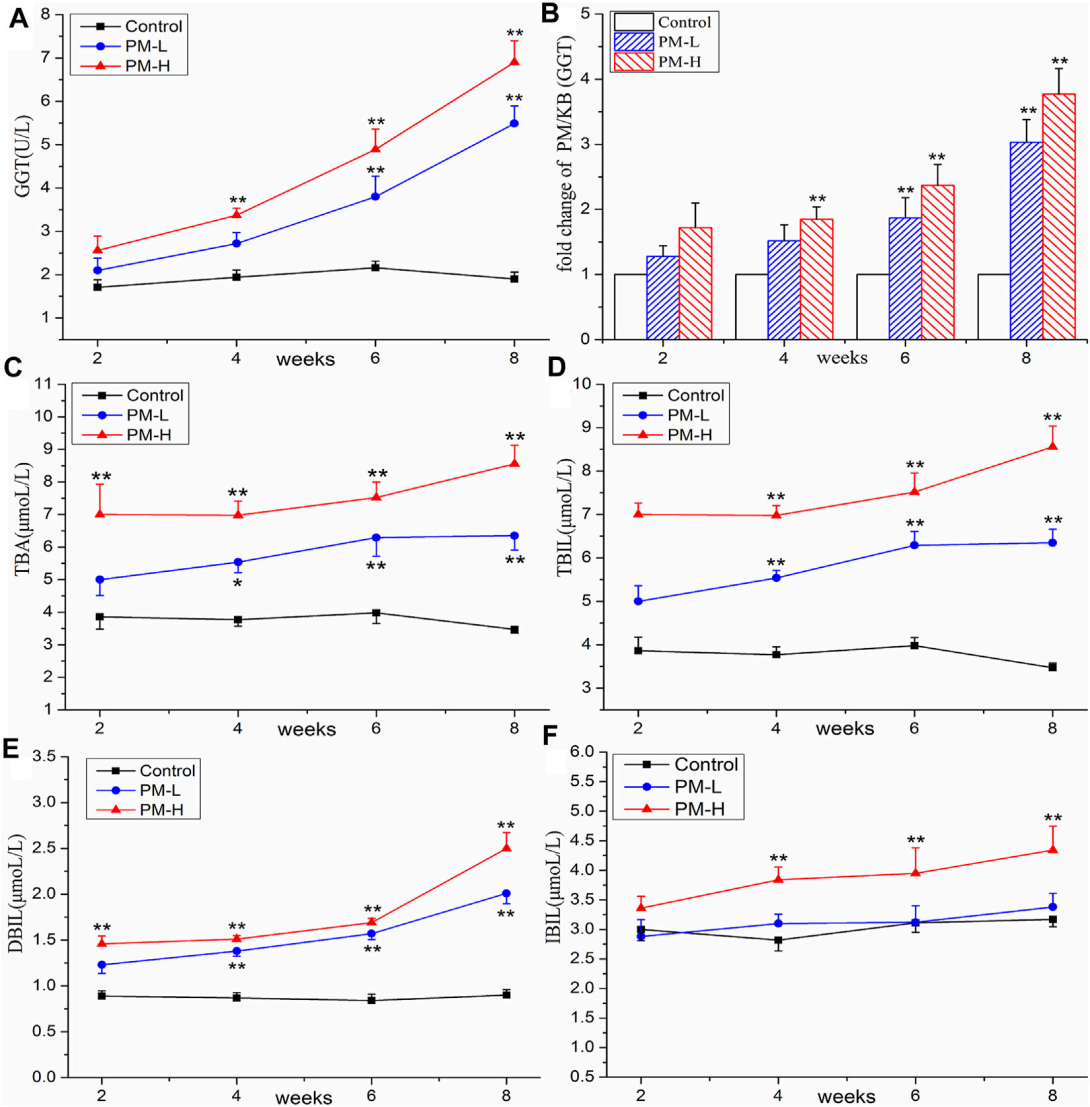
Contents of GGT **(A)**, TBA **(C)**, TBIL **(D)**, DBIL **(E)**, IBIL **(F)** and change of GGT **(B)** in control, PM-L and PM-H group at the end of second, fourth, sixth, and eighth week after administration. (mean ± SEM, *n* = 8, *p < 0.05, **p < 0.01).

The results of the biochemical analysis are shown in Figures 2, 3. Compared with the control group, all serum indices of liver injury showed an increasing trend and reached a significant difference in the eighth week. The levels of AST (Figure 2A) and ALT (Figure 2B) increased significantly only in the high-concentration group from the fourth week, and AST/ALT ratio (Figure 2C) did not change significantly, indicating that the damage to the hepatocyte type was not very serious. It is worth noting that the levels of DBIL (Figure 3E), ALP (Figure 2D) and TBA (Figure 3C) began to increase significantly in the second week, and that of IBIL (Figure 3F) and TBA (Figure 3C) began to increase markedly in the fourth week. What is more, it should be noted that ALP level was nearly twice that of the normal group at the eighth week (Figure 2E), and the ratio of ALT/ALP (R) (Figure 2F) was less than two [R= (ALT measured value/ALT upper limit of normal value)/(ALP measured value/ALP upper limit of normal value)]. TBA content also significantly increased by more than two times in the eighth week, TBIL content also increased by nearly two times, and the level of GGT (Figures 3A, B) showed a nearly 3-fold increase. A significant increase in these indices is usually related to cholestatic liver injury.

### 3.3 Effect of PM on histopathology in liver tissue

**FIGURE 4:**
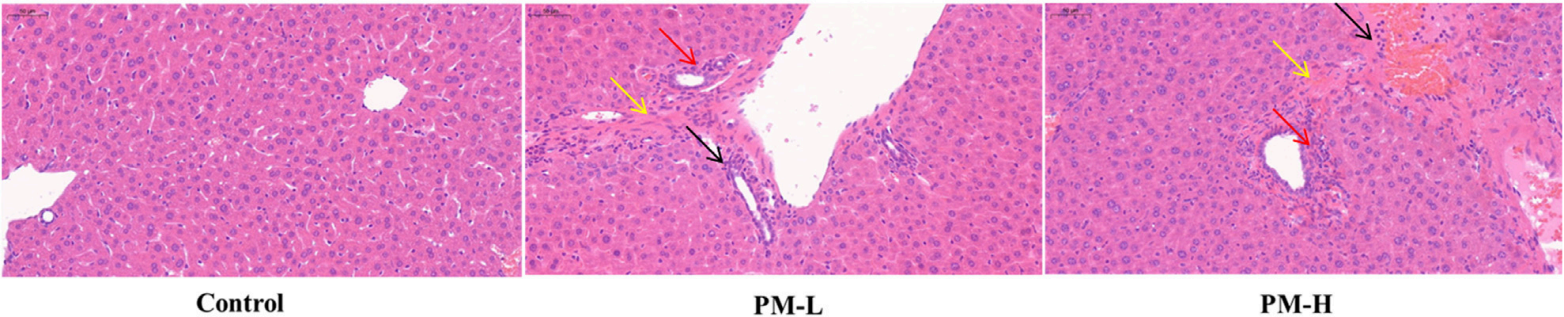
Liver histology (H&E stain, 200x, Scale bar = 50 µm) of mice; Hepatocyte necrosis and dissolution, replaced by hyperplastic connective tissue (yellow arrow), Inflammatory cell aggregation (black arrow), Peribiliary injury (red arrow).

Histopathological observations are shown in Figure 4. Under a ×200 visual field, the liver plates in the blank group were arranged regularly and orderly, the hepatic sinuses were not significantly expanded or squeezed, there was no obvious abnormality in the portal area between adjacent hepatic lobules, and no obvious inflammatory changes were found. In the administration group, with an increase in dosage, inflammatory cell infiltration and aggregation, hepatocyte swelling, and hepatocyte necrosis increased in the portal area. In particular, the bile duct cells around the bile duct were seriously damaged. Liver staining showed long-term administration of PM caused pathological changes in the liver.

### 3.4 Effect of PM on bile acids composition of liver and gallbladder

The extraction ion chromatograms of bile acids in the liver and gallbladder samples are shown in the Supplementary Material. The six groups of bile acid isomers were well separated under the established chromatographic conditions. The established quantitative method of bile acid has been inspected and verified by methodology, and the results of methodology inspection are in the additional materials.

**FIGURE 5:**
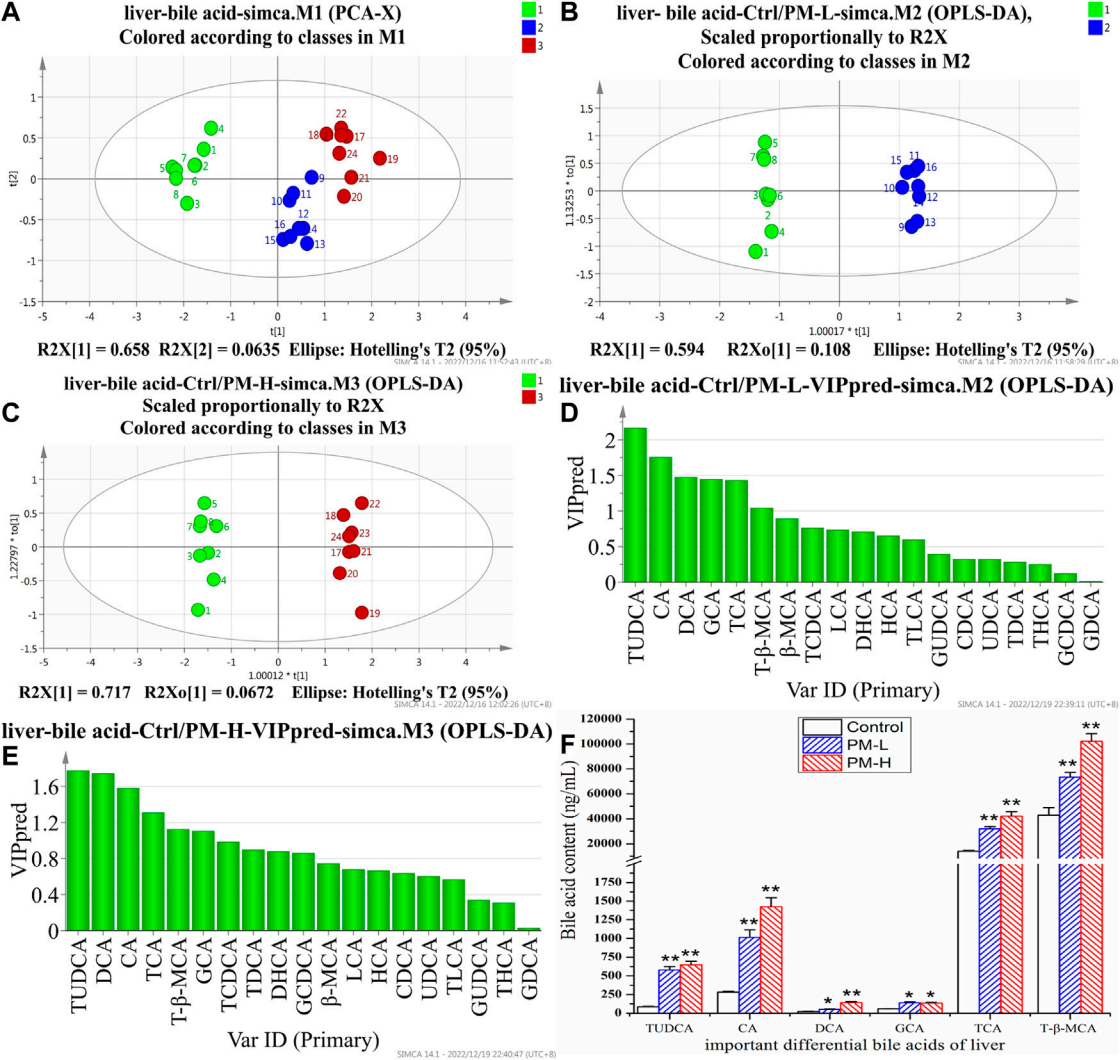
Score chart of PCA analysis of 19 bile acids in liver **(A)**; score chart and VIP values of OPLS-DA analysis of 19 bile acids in liver **(B–E)**; The screened important differential bile acids and their contents **(F)**. (X± SEM, *n* = 8, *p < 0.05, **p < 0.01).

**FIGURE 6:**
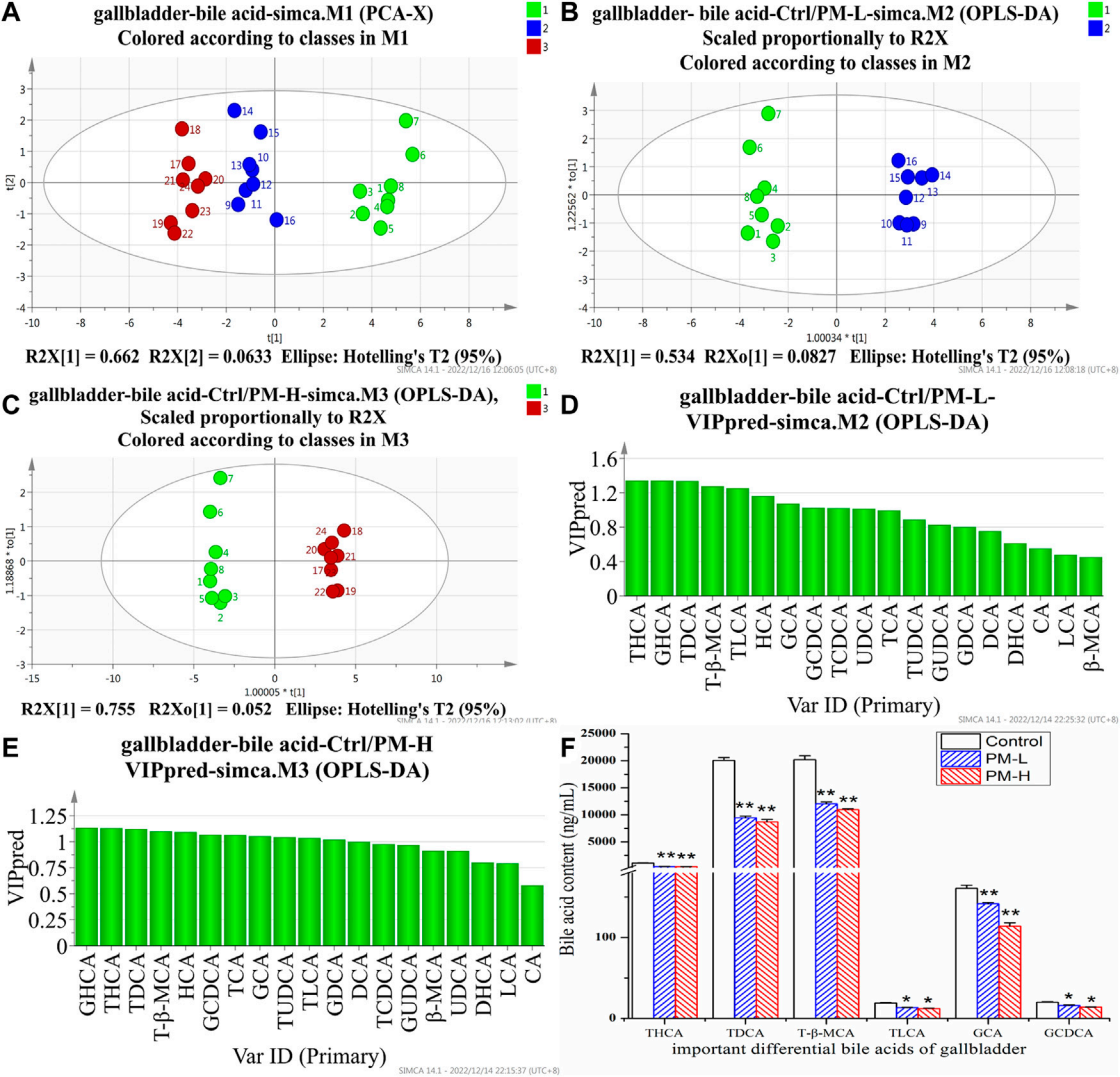
Score chart of PCA analysis of 19 bile acids in gallbladder **(A)**; core chart and VIP values of OPLS-DA analysis of 19 bile acids in liver **(B–E)**; The screened important differential bile acids and their contents **(F)**. (X± SEM, *n* = 8, *p < 0.05, **p < 0.01).

After 8 weeks of administration, we used the HPLC-QQQ-MS/MS method and observed that the content of bile acid in the liver tended to increase, while that in the gallbladder tended to decrease. We further used PCA (Figures 5A, 6A) and OPLS-DA (Figures 5B, C, 6B, C) to identify important differential bile acids (With VIP > 1 and *p* < 0.05 as the discriminant criteria). As seen from the PCA plots, the PM-H, PM-L and Control group samples were able to be obviously separated and gathered separately. OPLS-DA analysis was performed screen out the latent variables for distinguishing between Control group and treatment group. The statistical results of bile acid content with significant differences in the liver and gallbladder were shown in Figures 5, 6 respectively.

We screened six significantly upregulate bile acids (TUDCA, CA, DCA, GCA, TCA, and T-β-MCA) in the liver (Figures 5D–F). The THCA, TDCA, T-β-MCA, TLCA, GCA, GCDCA and TCDCA contents in the gallbladder (Figures 6D–F) were also markedly lower than those in the control group. The detection results for bile acids in the gallbladder also indirectly verified the detection results in the liver. These findings indicate that bile acid accumulates in the liver. The increase in free bile acid indicates that the synthesis of bile acid may be upregulated, and the increase in conjugated bile acid may be caused by the obstruction of bile acid efflux. These results not only provide us with evidence that PM leads to cholestasis, but also provide us with direction to discover the characteristics and mechanisms of PM induced liver injury.

### 3.5 Effects of PM on target gene transcription and protein expression

**FIGURE 7:**
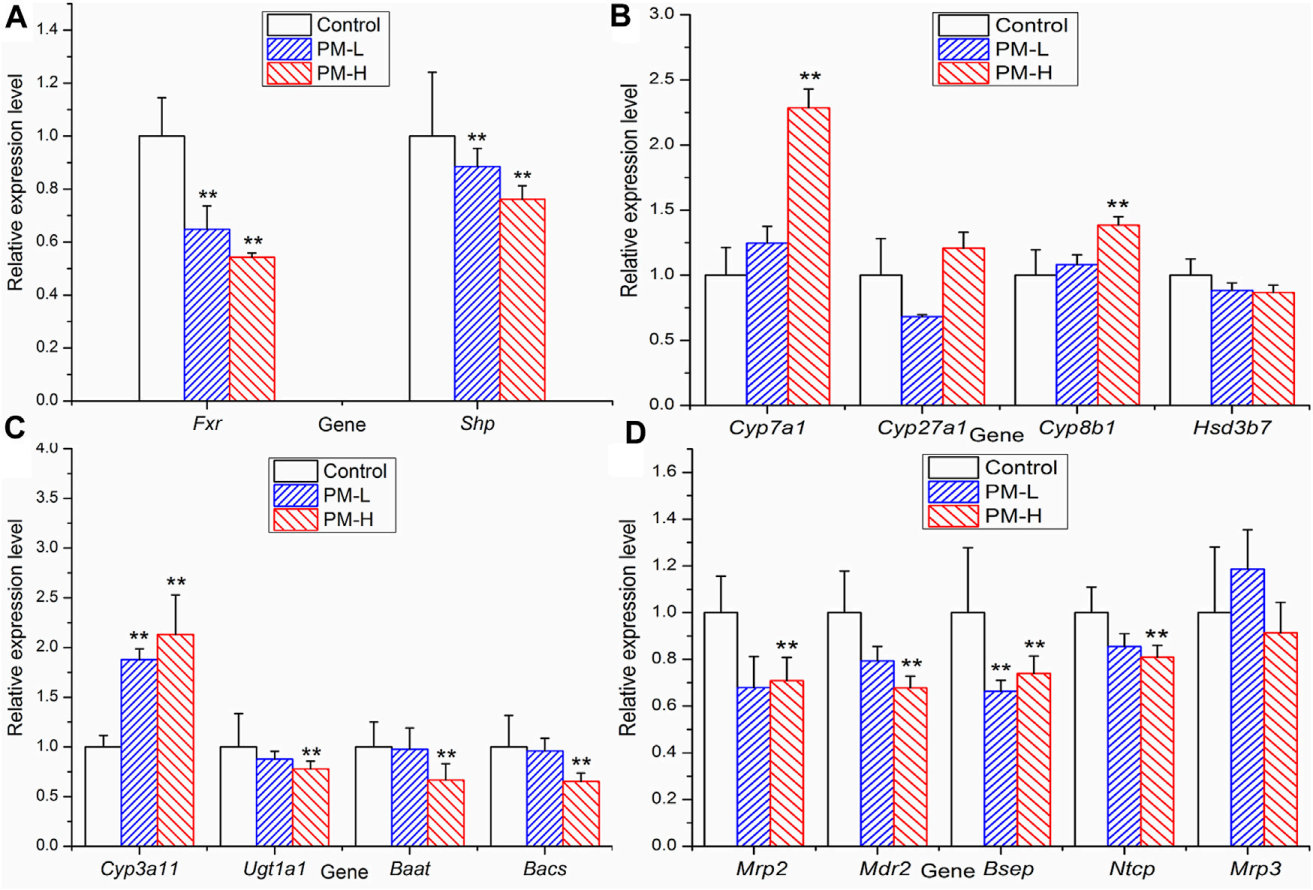
Relative expression of transcription factor **(A)**, metabolic enzyme **(B, C)** and transporters **(D)** genes in mice after 8 weeks administration (X ± SEM, *n* = 6, *p < 0.0, **p < 0.01).

In order to further study which important links in the process of bile acid metabolism are affected by polygonum multiflorum extract, we detected the mRNA level and protein expression of regulatory proteins in bile acid metabolism process. Figure 7 shown that after 8 weeks of administration, the levels of transcription factor *Fxr* and *Shp* were significantly decreased compared with those of the control group (Figure 7A). The metabolic enzyme levels of *Cyp7a1*, *Cyp27a1* and *Cyp3a11* were markedly increased and the levels of *Ugt1a1* and *Bacs* significantly decreased in the PM-H group, whereas those of *Cyp8b1*, *Hsd3b7* and *Baat* were not significant different from those in control group (Figures 7B, C). The transporter levels of *Bsep*, *Mrp2* and *Mdr2* were markedly decreased (Figure 7D), and the level of *Mrp3* was not significantly different from that in the control group.

**FIGURE 8:**
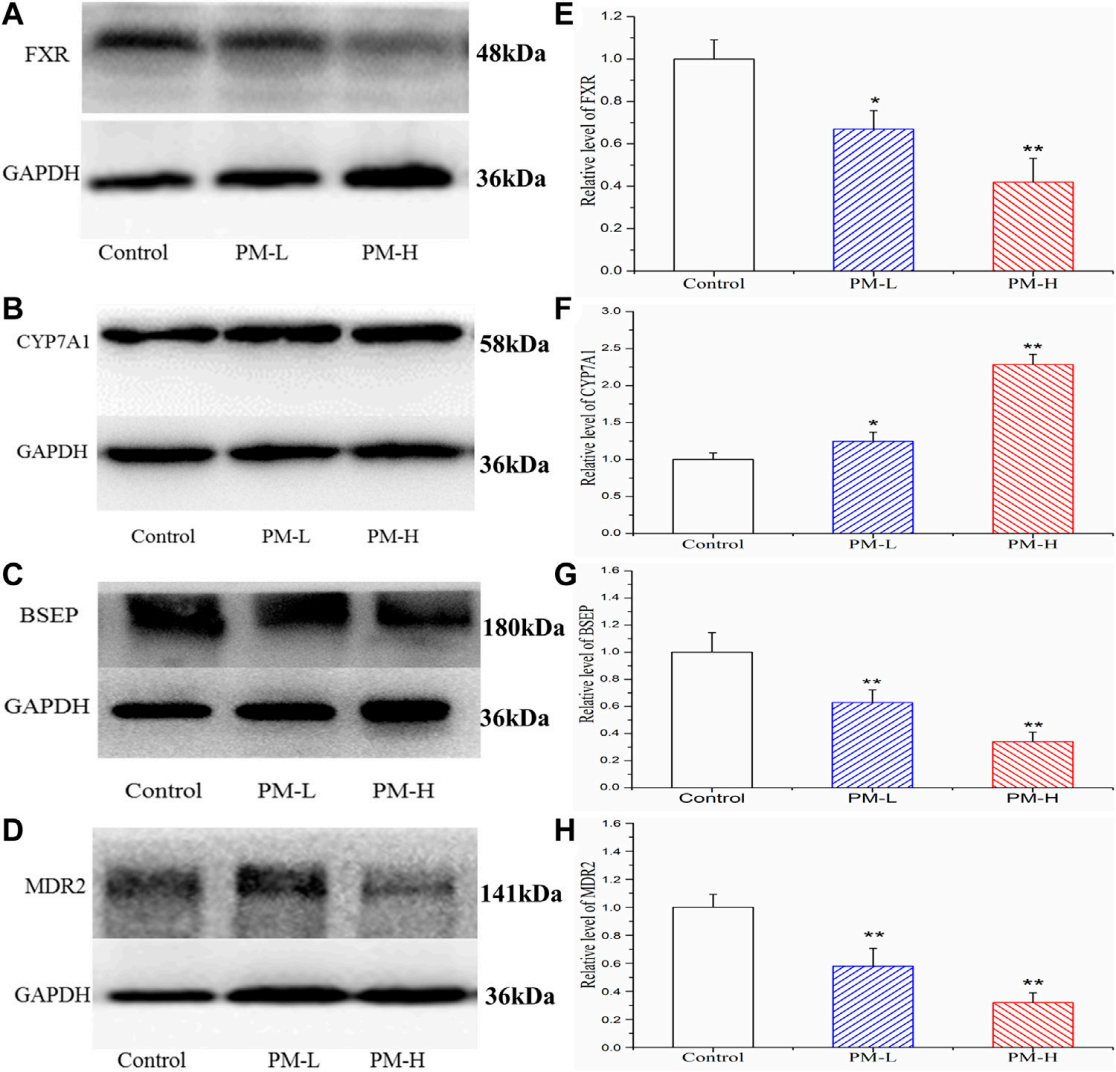
Effect of the protein expression of FXR **(A, E)**, CYP7A1 **(B, F)**, BSEP **(C, G)** and MDR2 **(D, H)** in mice after PM-L and PM-H treatment for 8 weeks. Quantification of the protein expression was performed by densitometric analysis of the blots following normalization to GAPDH expression. (mean ± SEM, *n* = 3, *p < 0.05, **p < 0.01).

Figure 8 showed the protein expression levels of transcription factors and metabolic enzymes with significant differences at the gene level. Compared to the control group, PM significantly decreased the protein expression of FXR (Figure 8A,E), BSEP (Figure 8C,G) and MDR2 (Figure 8D,H), while markedly increased the expression of CYP7A1 (Figure 8B,F).

Based on the above-mentioned experimental results and literature, we further detected the mRNA expression of transcription factors, metabolic enzymes, and transporters related to bile acid synthesis and transport using RT-qPCR. We further found significant differences in some proteins at the transcriptional level, such as upregulated *Cyp7a1*, downregulated *Fxr*, *Bsep*, *Mdr2*, etc. These results suggest that PM may cause cholestatic liver injury by inhibiting the expression of *Fxr*. However, *Cyp3a11* and *Mrp3* were highly expressed, which is conducive to the hydroxylation and efflux of bile acids. This may be due to the negative feedback regulation of the body when there are too many bile acids. According to the results of RT-qPCR, we selected the protein targets with significant effects of PM on the regulation of bile acid metabolism and transport and further verified the expression of these proteins using Western blotting.

## 4 Discussion

Reports on liver injury of PM mainly include the inherent toxic substance hypothesis and the specific heterogeneous liver injury hypothesis. Most of the studies on liver injury of PM focus on hepatocyte injury, while few studies focus on cholestatic hepatitis type. However, among the liver injury cases caused by PM, many patients have cholestatic symptoms, jaundice, and anorexia. Therefore, we analyzed the bile acid metabolism to explore the mechanism of cholestasis induced by PM.

Studies of PM-induced cholestatic liver injury are usually based on short-term experiments using lipopolysaccharide-induced models, though lipopolysaccharide also leads to cholestasis. At the same time, the design of short-term experiment was also different from the median time of liver injury induced by PM. What is more, Transient false positive changes in biochemical indicators of fitness may occur in short-term toxic exposure tests. In addition, Alcohol extract of PM was often used as the research object in the previous studies on liver injury. Alcohol extracts often exhibit hepatocellular liver damage. Given these findings, we took the aqueous extract of PM, the traditional method of use, as the research object, and conducted a relatively long animal administration experiment. The biochemical indices of animal serum were detected by dynamic detection method to prevent false positive liver injury caused by short-term adaptive increase of liver injury indices. Meanwhile, combined with the mouse model, mice are closer to the structure of the human hepatobiliary system. Both have a gallbladder structure that can better simulate the toxic effects of drugs on the hepatobiliary system.

In this experiment, with the increase in administration time of PM, the biochemical indices of animal serum increased significantly. In particular, GGT, ALP, TBA, TBIL and DBIL, which have important correlations with cholestasis, showed differences earlier than AST and ALT, indicating that liver injury induced by PM may cause changes in bile components such as bile acid and bilirubin in the early stage and become an important inducer of further liver injury. The elevation of ALP and GGT value are the most characteristic early manifestation of cholestasis. The guidelines for drug-induced liver injury in 2011 recommend this index of cholestatic liver injury as follows: The value of ALP is 2-fold higher than the ULN (normal value) and R ≤ 2 [R = (ALT measured value/ALT upper limit of normal value)/(ALP measured value/ALP upper limit of normal value)](Aithal et al., 2011). China’s guidelines for the diagnosis of cholestatic liver injury recommend that liver biochemical examination corresponding to ALP value > 1.5-fold ULN and GGT value >3-fold ULN can be diagnosed as cholestatic liver disease (Chinese Society of Hepatology, Chinese Society of Gastroenterology, and Chinese Society of Infectious Diseases of the Chinese Medical Association, C. M., 2015). In our experiment results, the R values, GGT value, and ALP value met the requirements of the guidelines. Therefore, it can be speculated that PM leads to cholestatic liver injury in mice.

After 8 weeks of administration, liver staining showed aggregation of inflammatory cells and necrosis of some hepatocytes in the liver, and the liver showed a certain trend of swelling. Especially in the portal area, the bile duct structure is obviously damaged. These means that the liver injury caused by PM is closely related to the damage of bile duct structure.

Bile acid metabolomics is a targeted metabolomics method that focuses on studying changes in the content and proportion of the bile acid pool in the body. It is one of the new techniques to study hepatobiliary diseases such as cholestasis and liver injury. This specific and sensitive MRM method can be used to study liver injury caused by bile acids. As bile acids are sterols composed of homologues and isomers with very similar structures, they contain conformational isomers with the same ion pair. The separation of conformational isomers is difficult using this method. Bile acids contain six groups of conformational isomers: 1) HDCA, CDCA, DCA, and UDCA; 2) GHDCA, GCDCA, GDCA, and GUDCA; 3) THDCA, TCDCA, TDCA, and TUDCA; 4) CA, β-MCA, and HCA; 5) TCA, T-β-MCA, and THCA; and 6) GCA and GHCA. It can be observed from the table that the six isomers were completely separated. Bile acid levels were confirmed using a single comparison. Since there is no reference substance for T-β-MCA, but T-β-MCA is also an important bile acid, we tried to use the standard curve of TCA to quantify the relative content of T-β-MCA. Bile acid is an endogenous substance that affects experimental results in methodological research. In this experiment, our reference method used the physical method of activated carbon adsorption to adsorb endogenous bile acids to prepare a blank matrix and establish an accurate quantitative method for bile acids (Bathena et al., 2013). We have referred to and improved the method and established an accurate quantitative method for bile acids.

We found that some conjugated bile acids (TUDCA, TCA, and T-β-MCA) had a significant upward trend in the liver, and were important differential bile acids to be screened in bile acid metabolomics. The intrahepatic increase of conjugated bile acids might be caused by the inhibition of bile acid transport. Then we also found that the content of CA, DCA and β-MCA of bile acid increased significantly. We located the synthesis process of bile acid. There were two main pathways in the synthesis process of bile acid, namely, the classical pathway and the alternative pathway. Based on the selected CA and DCA, we inferred that PM mainly affected the classical pathway of bile acid synthesis. These processes are regulated by FXR, so we need to focus on and verify key proteins downstream of FXR and FXR regulation in subsequent experiments. These results provide some references and indications for us to find out the causes of bile acid metabolism disorders, and can be mutually verified with the subsequent Q-PCR and WB results.

These results not only provide us with evidence that PM leads to cholestasis, but also provide us with inspiration to discover the characteristics and mechanisms of PM induced cholestatic liver injury. These findings indicate that bile acid accumulates in the liver, the increase of free bile acid indicates that the synthesis of bile acid may be upregulated, and the increase of conjugated bile acid may be caused by the obstruction of bile acid out flow.

In general, most bile acids in the body exist in the form of conjugated and maintain dynamic balance. Accumulation of hydrophobic bile acids in the liver has always been considered the main cause of liver injury in patients with cholestatic liver disease (Garcia-Canaveras et al., 2012). It has been reported that DCA can promote the polarization of M1 macrophages and the production of pro-inflammatory cytokines in a dose-dependent manner, resulting in inflammatory damage to the liver (Wang et al., 2020). In fact, when the bile component is abnormal or bile flow is reduced, the bile acids may become more harmful to the liver because of the prolonged contact between the bile component and the membrane of hepatocytes or bile duct cells (Pean and Tordjmann, 2014). Recent studies have also found that the accumulation of some conjugated bile acids can also promote liver fibrosis and even the development of liver cancer (Jia et al., 2018).

Bile acids are a series of endogenous cholesteranes that are synthesized by cholesterol in the liver. Bile acid is produced by cholesterol as a raw material under the regulation of CYP7A1, CYP8B1, and CYP27A1, etc. and is metabolized into bound bile acids through BACS, BAAT, and SULT2A1, etc. Bile acids discharged from the hepatocytes and enter the bile duct and blood from the bile duct and hepatocyte membrane through BSEP, MRP2/3/4, and MDR2, respectively. Under normal circumstances, the bile acid metabolism is balanced. However, cholestasis caused by blocking the metabolism and excretion of bile acids can cause functional damage to the liver, mitochondrial damage, apoptosis, and hepatocyte failure (Hofmann, 1999). MDR2 deficient mice are an animal model showing obvious cholangitis and cholestasis, and their prognosis produces a human-like liver cancer phenotype (Miethke et al., 2016). The loss and inhibition of BSEP can lead to cholestatic liver injury (Soroka and Boyer, 2014). Inhibition of MRP2 leads to further occurrence and development of cholestasis (Wu et al., 2018). Inhibitions of BACS and BAAT lead to further cholestasis (Pircher et al., 2003; Clayton, 2011). The literature in the field of bile acids physiology has also experienced great expansion, mainly due to the discovery of bile acids nuclear receptors, especially the farnesoid X receptor (FXR) and membrane-bound bile acids receptor five (TGR5) (Porez et al., 2012). FXR is an important nuclear transcription factor that regulates bile acid metabolism. It can regulate the metabolic homeostasis of bile acids in various ways (Thompson et al., 2018). It is a ligand-activated transcription factor belonging to the nuclear receptor superfamily. It plays an important regulatory role in biochemical reaction pathways such as bile acids, carbohydrates, and lipid metabolism. FXR is highly expressed in the liver, intestine, and kidney, and plays an important role in liver diseases. Liver injury caused by cholestasis is caused by bile acid metabolism disorders. Previous studies have suggested that when FXR function is inhibited, the liver shows bile acid accumulation, which produces toxic reactions. The reasons can be summarized as follows. After FXR is inhibited, the expression of downstream target genes changes, which then regulates the increase of bile acid synthase CYP7A1, upregulates bile acid uptake transporters, and downregulates bile acid efflux transport (Garzel et al., 2014). After a series of reactions, the liver ingests excessive bile acid which cannot be excreted, and the accumulation of bile acids in the liver produces hepatotoxicity. β-MCA, UDCA, and their taurine/glycine conjugates are FXR antagonists. The transcriptional activity of FXR can also be inhibited by upregulating the levels of these bile acids. In contrast, other bile acids, such as CDCA, are agonists of FXR. The interaction between agonists and inhibitors also regulates the metabolic and secretory balance of bile acids. Our experimental results showed that after administration of PM, the content of T-β-MCA, UDCA, TUDCA and β-MCA increased significantly in the liver, which further led to the inhibition of FXR.

Based on the above-mentioned experimental results and literature, we further detected the mRNA expression of transcription factors, metabolic enzymes, and transporters related to bile acid synthesis and transport using RT-qPCR. We further found significant differences in some proteins at the transcriptional level, such as upregulated *Cyp7a1*, downregulated *Fxr*, *Bsep*, *Mdr2*, etc. These results suggest that PM may cause cholestatic liver injury by inhibiting the expression of *Fxr*. However, *Cyp3a11* and *Mrp3* were highly expressed, which is conducive to the hydroxylation and efflux of bile acids. This may be due to the negative feedback regulation of the body when there are too many bile acids. According to the results of RT-qPCR, we selected the protein targets with significant effects of PM on the regulation of bile acid metabolism and transport and further verified the expression of these proteins using Western blotting.

In this study, we found that PM can cause disorders in bile acid metabolism by disturbing bile acid metabolism and excessive bile acid accumulation in the liver, causing liver injury. The results of bile acid metabolomics and biochemistry revealed that hepatotoxic bile acids were increased. Further studies on the regulation of bile acids using molecular biology found that PM interfered with the process of bile acid metabolism and the main regulatory targets. PM inhibits the protein expression of bile acid transport to bile duct, resulting in obstruction of bile outflow. It also affects the mRNA levels of key enzymes in bile acid metabolism, particularly by inhibiting the binomial metabolism of bile acid and promoting the synthesis of bile acid. These effects may lead to excessive accumulation of bile acids in hepatocytes, resulting in liver injury. According to the literature, we can further speculate that bile acids cannot be fully discharged into the gallbladder, resulting in the failure of bile flow in the gallbladder, which is further discharged from the gallbladder to the small intestine. The decrease in bile acids in the small intestine further reduces the activation of the intestinal FXR receptor, and then reduces the inhibition of the FXR-FGF15 pathway on bile acid intrahepatic synthase CYP7A1, resulting in an increase in bile acid intrahepatic synthesis.

**FIGURE 9:**
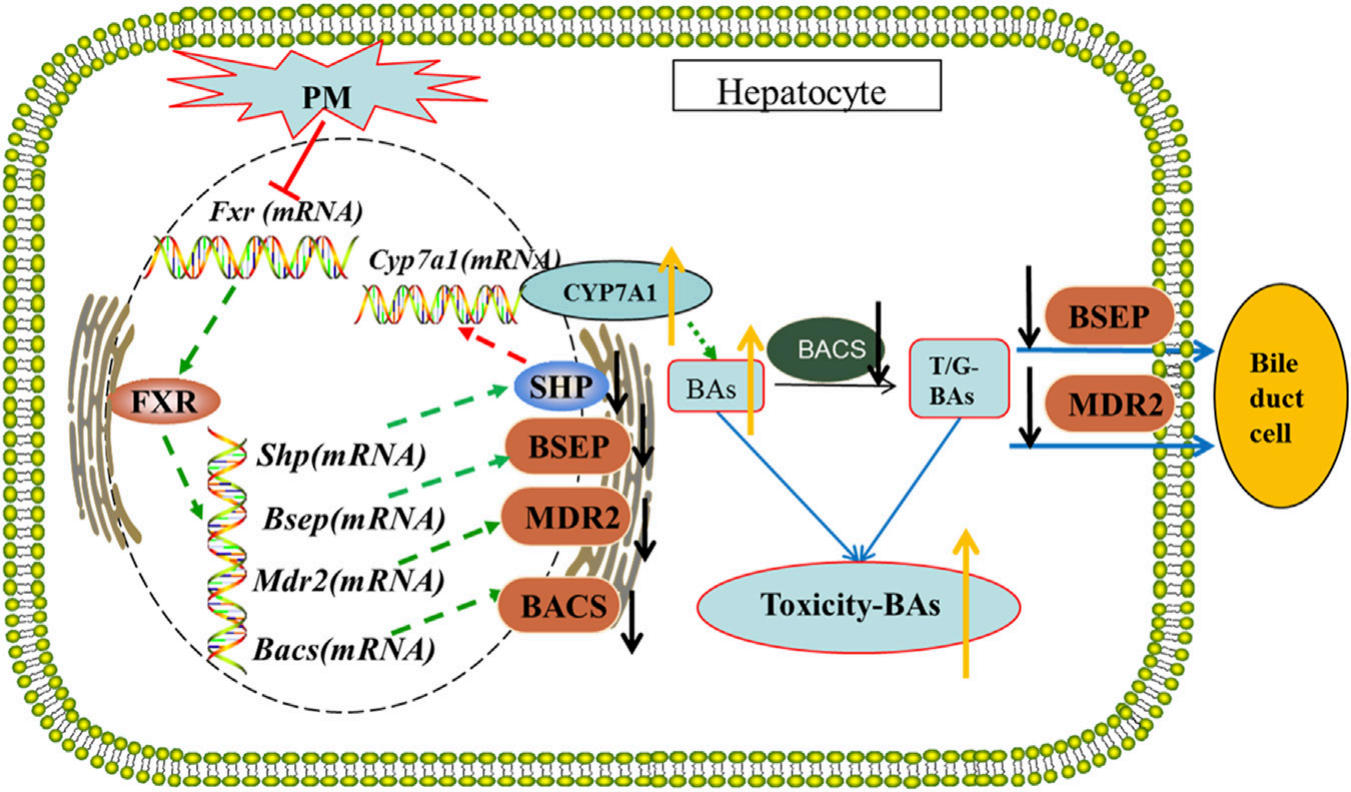
The possible mechanism of cholestatic liver injury by PM.

In summary, PM can inhibit the expression of FXR in the liver, resulting in a decrease in the expression of the BSEP and MDR2 and an increase in CYP7A1 which eventually leads to an increase in bile acid production and the obstruction of bile acid efflux, resulting in the accumulation of bile acid in the liver, leading to cholestatic liver injury. PM affects the transport and synthesis pathway of bile acids in the liver and destroys the steady-state environment of bile acids in the body, which may be the main mechanism of cholestatic hepatotoxicity induced by PM. The possible mechanisms underlying cholestatic liver injury is shown in Figure 9.

This study mainly focused on the changes in bile acid-metabolizing enzymes and transporter expression to explain the possible mechanism of liver toxicity of PM, but also ignored the direct effect of PM on the transport capacity of transporters. In the future laboratory, to investigate the mechanism of liver toxicity more comprehensively, we can consider conducting vesicular transport test to study whether PM affect the transport capacity of bile acid transporters in the later period. Since this is more of an early stage exploratory study and we are complying with the 4R rules (Reduce, refine, replace- responsibility), so we only set up two dosing groups. In addition, bile acid is currently considered a key link in the unclear pathway between intestinal microbiota and liver pathophysiology (Islam et al., 2011). Therefore, this study can further study the mechanism and effect of PM-intestinal flora-bile acid-liver injury. We hope that this idea and method can provide a new perspective on the mechanism of liver injury caused by PM.

## 5 Conclusion

By inhibiting the transcription and translation of FXR, PM inhibits the expression of BSEP and MDR2 and promotes the expression of CYP7A1, resulting in a decrease in the efflux of bile acids and an increase in the synthesis of bile acids, thus deviating from the stable state, and causing cholestatic liver injury.

## Data Availability

The original contributions presented in the study are included in the article/Supplementary Material, further inquiries can be directed to the corresponding author.
