# Supplementary material for: Polygoni Multiflori Radix interferes with bile acid metabolism homeostasis by inhibiting Fxr transcription, leading to cholestasis
Source: Front Pharmacol. 2023 Mar 6;14:1099935. doi: 10.3389/fphar.2023.1099935 (PMC10025474; doi:10.3389/fphar.2023.1099935)

Supplementary Material - Content determination and fingerprinting of PM extract

**1 Content determination**

Detection methods: agilent high-performance liquid chromatograph-ultraviolet multiwavelength detector was used, and the chromatographic column was Agilent Poroshell 120 EC-C18 (50mmx4.6,5um). The mobile phases were methanol (B) and 0.1% formic acid aqueous solution (A). The gradient elution procedure was as follows:0-8min,10%-35%B;8-11min,35%-55%B;11-13min,55%-55%B;13-15min,55%-70%B;15-19min,70%-90%B,19-25min,90%-90%B。Flow rate: 1ml/min; Detection wavelength: 254nm; Column temperature: 35℃; the injection volume was 2 µL.


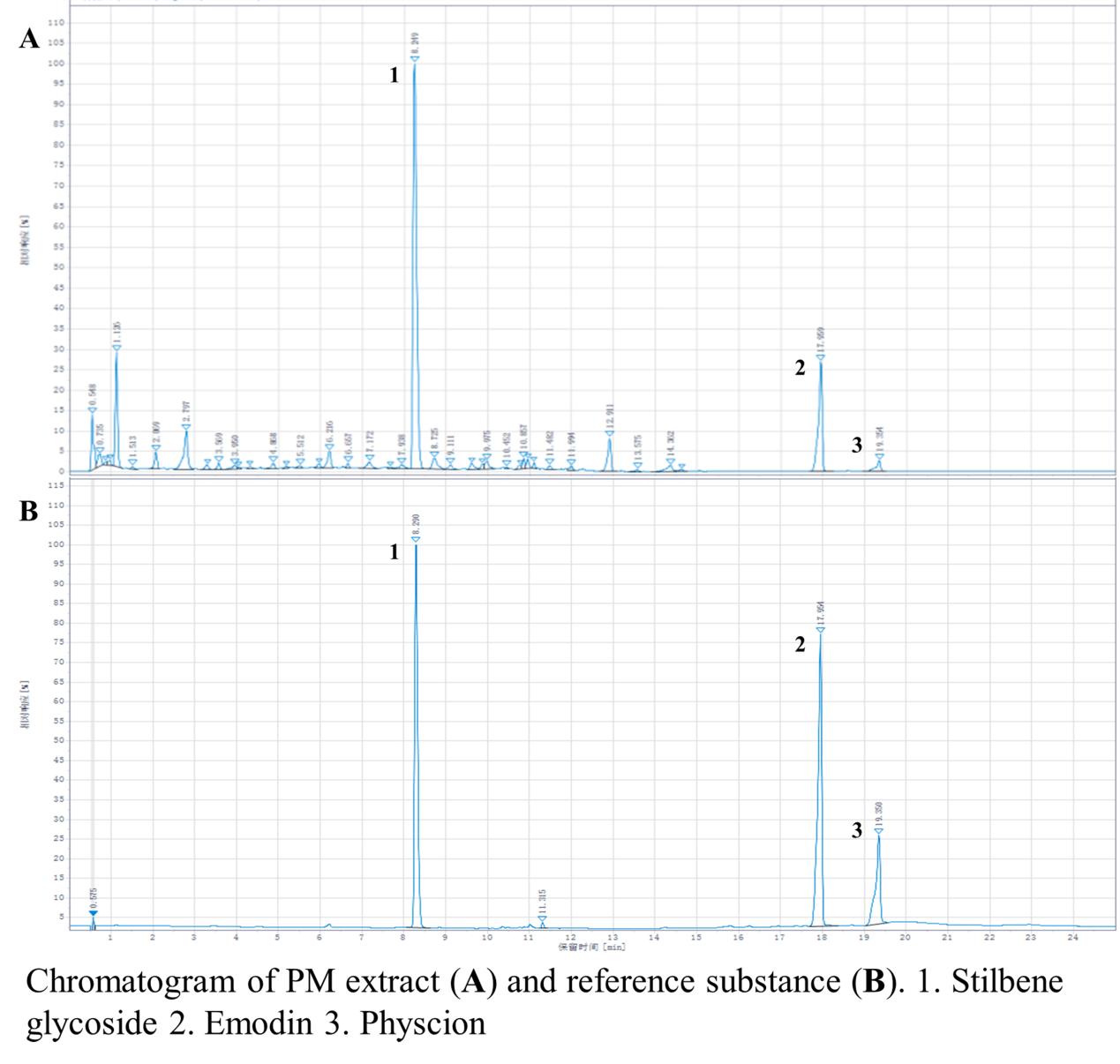


**2 Fingerprint**

Detection methods: agilent high-performance liquid chromatograph-ultraviolet multiwavelength detector was used, and the chromatographic column was Agilent Poroshell 120 EC-C18 (50mmx4.6, 5um). The mobile phases were methanol (B) and 0.1% formic acid aqueous solution (A). The gradient elution procedure was as follows:0-8min,10%-35%B;8-11min,35%-55%B;11-13min,55%-55%B;13-15min,55%-70%B;15-19min,70%-90%B,19-25min,90%-90%B。Flow rate: 1ml/min; Detection wavelength: 254nm，280nm and 320nm; Column temperature: 35℃; the injection volume was 2 µL.


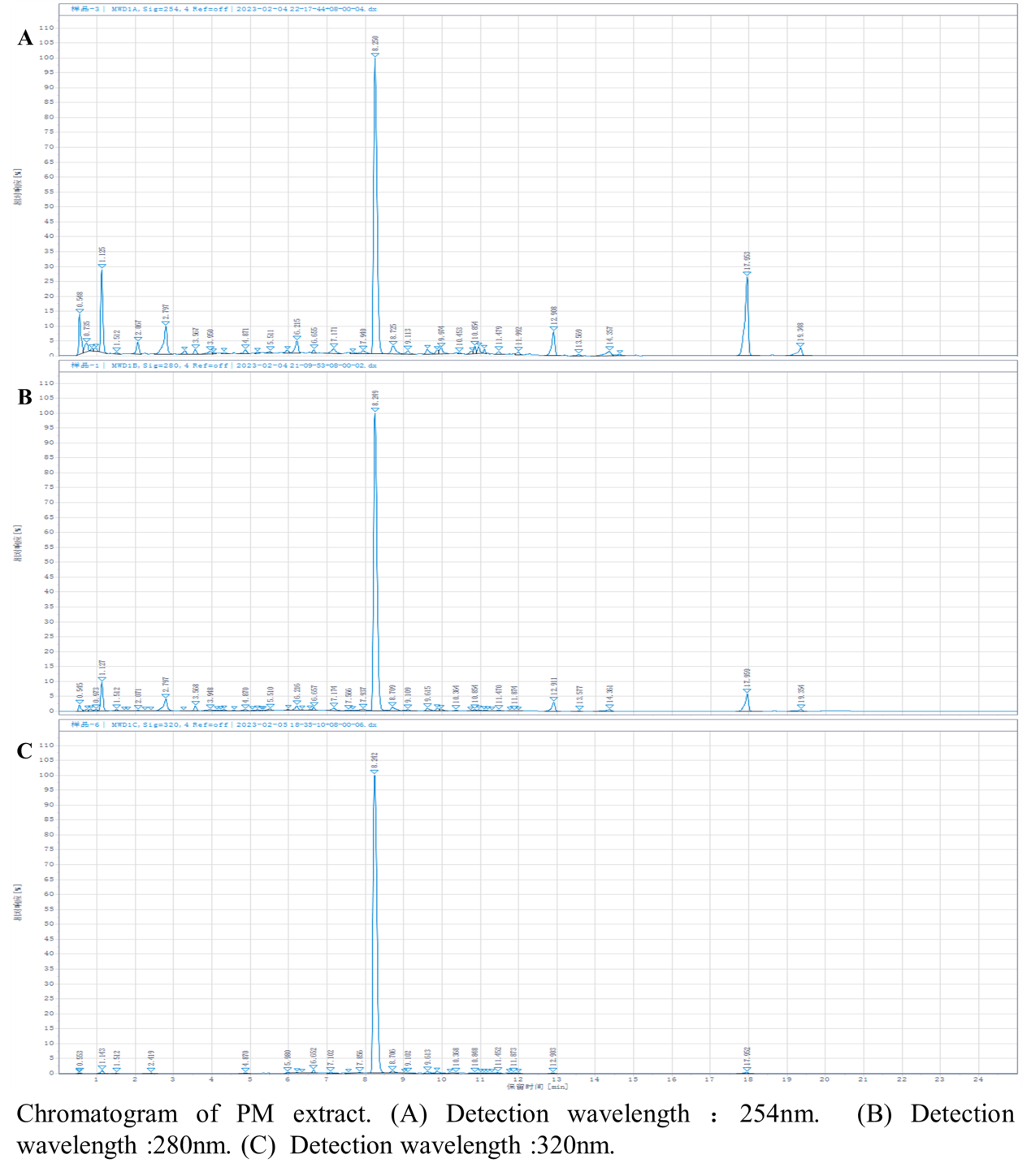

Supplement: Supplementary file 1 [file DataSheet4.DOCX]
