# Supplementary material for: Polygoni Multiflori Radix interferes with bile acid metabolism homeostasis by inhibiting Fxr transcription, leading to cholestasis
Source: Front Pharmacol. 2023 Mar 6;14:1099935. doi: 10.3389/fphar.2023.1099935 (PMC10025474; doi:10.3389/fphar.2023.1099935)
Supplement: Supplementary file 2 [file DataSheet3.DOCX]

Supplement- Target identification of anthraquinones and stilbene glycosides in the water extract of PM based on UHPLC-Q-TOF-MS/MS

1 Chromatogram of UHPLC-Q-TOF-MS/MS

2 Table of anthraquinones and stilbene glycosides

| **Type of compound** | **name of compound** | **molecular formula** | **additive method** | **theoretical molecular weight** | **measured molecular weight** | **relative error**  **(ppm)** | **retention time** | **Secondary fragment ion** |
| --- | --- | --- | --- | --- | --- | --- | --- | --- |
| **Anthra**  **Quinones** | Physcion | C_16_H_12_O_5_ | [M-H]^-^ | 283.0612 | 283.0617 | 1.78 | 29.629 | 240.0414,  212.0462 |
|  | Modin-8-O-β-D-glucopyranoside | C_21_H_20_O_10_ | [M-H]^-^ | 431.0984 | 431.0985 | 0.3 | 27.617 | 269.0429,  197.057 |
|  | Rhein | C_15_H_8_O_6_ | [M-H]^-^ | 329.0303 | 329.0304 | 0.33 | 23.543 | 314.0047 |
|  | Emodin | C_15_H_10_O_5_ | [M-H]^-^ | 269.0455 | 269.0464 | 3.17 | 39.339 | 241.0484 |
| **Stilbene glycosides** | (E)-2,3,5,4ʹ-Tetrahydroxystilbene-  2,3-O-diglucoside | C_26_H_32_O_14_ | [M-H]^-^ | 567.1719 | 567.1720 | 0.12 | 24.108 | 528.7985 |
|  | Trans-2,3,5,4 '-tetrahydroxystilbene-  2-O-glucosyl-(1→6)-β-D-glucoside | C_26_H_32_O_14_ | [M-H]^-^ | 567.1719 | 567.172 | 0.12 | 22.811 | 521.2029,  549.1310 |
|  | Cis-2,3,5,4'-tetrahydroxystilbene-  2-O-glucosyl-(1→6)-β-D-glucoside | C_26_H_32_O_14_ | [M+COOH]^-^ | 613.1774 | 613.1782 | 1.29 | 23.426 | 567.1716 |
|  | (E)-2,3,5,4ʹ-Tetrahydroxystilbene-  2-O-(2ʹʹ-O-feruliacyl)-β-D-glucoside | C_30_H_30_O_12_ | [M-H]^-^ | 581.1664 | 581.1663 | 0.26 | 26.869 | 419.1144 |
|  | (E)-2,3,5,4ʹ-Tetrahydroxystilbene-2-O-  (2ʹʹ-O-P-hydroxybenzoyl)-β-D-glucoside | C_27_H_26_O_11_ | [M-H]^-^ | 525.1402 | 525.1401 | 0.26 | 26.27 | 269.0471,  469.0267 |
|  | (E)-2,3,5,4ʹ-Tetrahydroxystilbene-2-O-  (2ʹʹ-O-galloyl)-β-D-glucoside | C_27_H_26_O_13_ | [M-H]^-^ | 557.1301 | 557.131 | 1.68 | 24.757 | 539.1225,  405.1196 |
|  | (E)-2,3,5,4ʹ-Tetrahydroxystilbene-2-O-  (3ʹʹ-O-galloyl)-β-D-glucoside | C_27_H_26_O_13_ | [M-H]^-^ | 557.1301 | 557.1309 | 1.5 | 25.305 | 405.118 |
|  | (E)-2,3,5,4ʹ-Tetrahydroxystilbene-2-O-  (6ʹʹ-O-galloyl)-β-D-glucoside | C_27_H_26_O_13_ | [M-H]^-^ | 557.1301 | 557.1307 | 1.14 | 25.921 | 444.276 |
|  | Resveratrol-3-O-β-D-(2″-O-galloyl)-  glucoside | C_27_H_26_O_13_ | [M-H]^-^ | 557.1301 | 557.1305 | 0.78 | 25.771 | 553.661 |
|  | (E)-2,3,5,4ʹ-Tetrahydroxystilbene-2-O-  (6ʹʹ-O-acetyl)-β-D-glucoside | C_22_H_24_O_10_ | [M-H]^-^ | 447.1297 | 447.1296 | 0.07 | 25.971 | 433.1153,  270.0529 |
|  | (E)-2,3,5,4ʹ-Tetrahydroxystilbene-2-O-  (2ʹʹ-O-acetyl)-β-D-glucoside | C_22_H_24_O_10_ | [M-H]^-^ | 447.1297 | 447.1299 | 0.51 | 25.355 | 433.1153,  242.0509 |
|  | (E)-2,3,5,4ʹ-Tetrahydroxystilbene-2-O-  β-α-rhamnoside | C_20_H_22_O_8_ | [M-H]^-^ | 389.1242 | 389.1247 | 1.31 | 25.838 | 243.0671 |
|  | 3,5,4ʹ-Trihydroxystilbene-3-O-  β-D-glucoside | C_20_H_22_O_8_ | [M-H]^-^ | 435.1297 | 435.1303 | 1.45 | 24.342 | 227.0701,  143.0498 |
|  | 3,5,4ʹ-Trihydroxystilbene-4ʹ-O-  β-D-glucoside | C_20_H_22_O_8_ | [M-H]^-^ | 435.1297 | 435.1291 | 1.31 | 25.737 | 227.0701,  185.0579 |
|  | (E)-2,4,6,4ʹ-Tetrahydroxystilbene-2-O-  β-D-glucoside | C_20_H_22_O_9_ | [M-H]^-^ | 405.1191 | 405.1199 | 1.96 | 23.593 | 243.0627,  137.0218 |
|  | (E)-2,3,5,4ʹ-Tetrahydroxystilbene-2-O-  β-D-glucoside | C_20_H_22_O_9_ | [M-H]^-^ | 405.1191 | 405.1202 | 2.7 | 24.541 | 243.0627,  137.0218 |
|  | (Z)-2,3,5,4ʹ-Tetrahydroxystilbene-2-O-  β-D-glucoside | C_20_H_22_O_9_ | [M-H]^-^ | 405.1191 | 405.1199 | 1.96 | 24.657 | 243.0627,  137.0218 |
