# Supplementary material for: Polygoni Multiflori Radix interferes with bile acid metabolism homeostasis by inhibiting Fxr transcription, leading to cholestasis
Source: Front Pharmacol. 2023 Mar 6;14:1099935. doi: 10.3389/fphar.2023.1099935 (PMC10025474; doi:10.3389/fphar.2023.1099935)
Supplement: Supplementary file 4 [file DataSheet1.DOCX]

**Supplement methodological investigation of bile acids in liver**

**1.Specificity and separation**

**Chromatogram of blank matrix with internal standard added**

**Ion chromatograms of extracts of various bile acids**

**
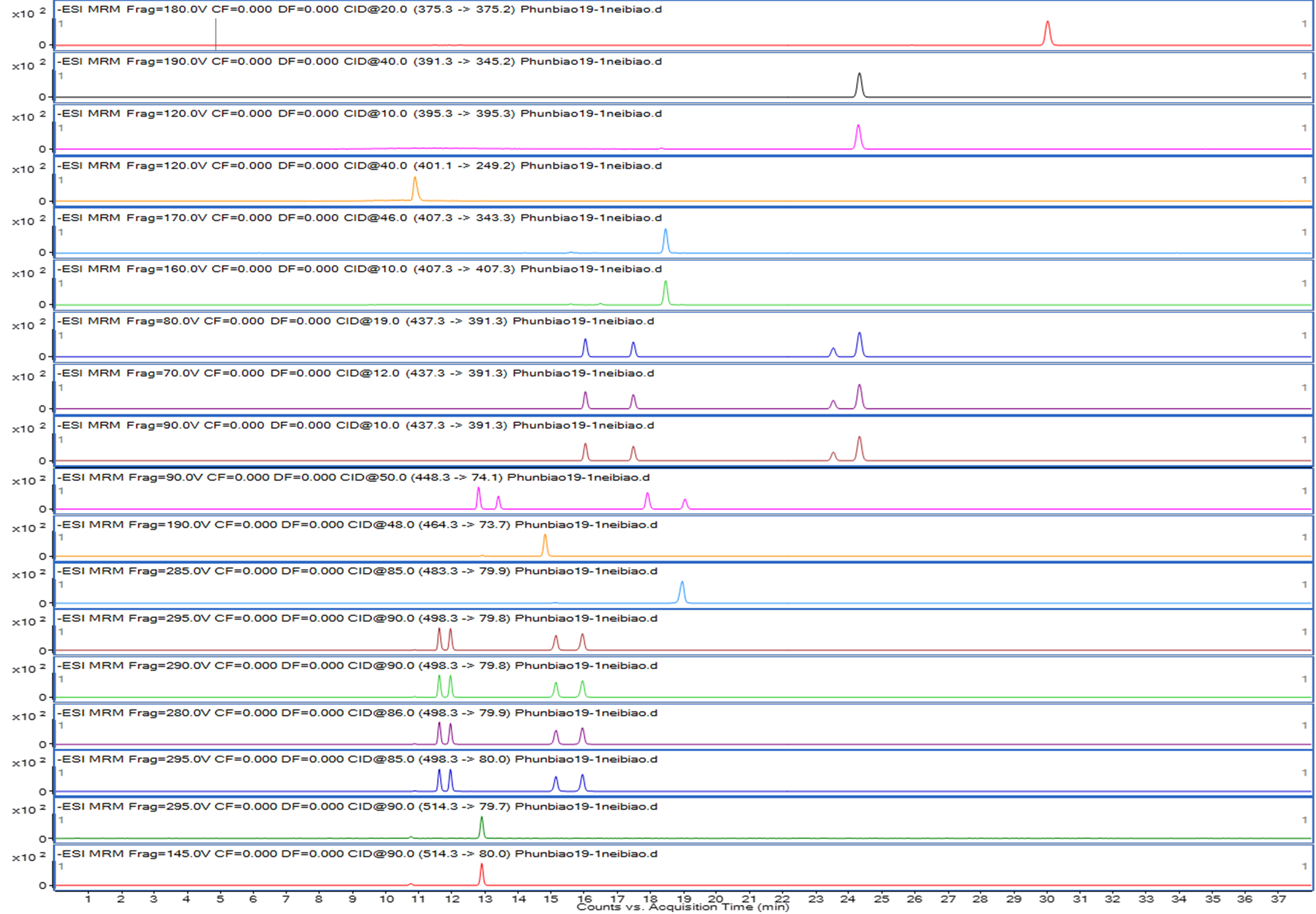
**

**2. linear range and correlation coefficient**.

The correlation coefficient meets the requirements of quantitative detection of biological samples, indicating that the calibration curve can be used for accurate quantitative analysis within the linear range. The results were shown in Tab 1.

Tab.1 Calibration curve and correlation coefficient of the 19 BAs.

| **Compound** | **Calibration curve** | **R^2^** | **Linear ranges** |
| --- | --- | --- | --- |
| LCA | y=0.1687*x-0.002486 | 0.999 | 2.44-625ng/ml |
| DCA | y=0.1655*x+0.00263 | 0.989 | 4.88-625ng/ml |
| DHCA | y=0.132*x-7.53*10^-5^ | 0.999 | 2.44-625ng/ml |
| CA | y=0.0485*x-2.6946*10^-5^ | 0.999 | 4.88-2500ng/ml |
| B-MCA | y=0.038943*x+2.563241*10^-4^ | 0.999 | 4.88-625ug/ml |
| UDCA | y=1.32*x-0.0015 | 0.999 | 4.88-625ng/ml |
| HCA | y=2.244*x-0.0098 | 0.999 | 4.88-625ng/ml |
| CDCA | y=0.4626*x+0.002051 | 0.999 | 4.88-625ng/ml |
| GUDCA | y=0.1015*x-1.755*10^-4^ | 0.999 | 2.44-625ng/ml |
| GHCA | y=0.1045*x+1.107*10^-4^ | 0.999 | 2.44-625ng/ml |
| GDCA | y=0.1656*x-2.436*10^-4^ | 0.999 | 2.44-625ng/ml |
| GCDCA | y=0.06*x-4,56*10^-5^ | 0.998 | 2.44-625ng/ml |
| GCA | y=0.0455*x-8.44*10^-5^ | 0.998 | 4.88-625ng/ml |
| TLCA | y=0.0408*x-7.425*10^-6^ | 0.999 | 2.44-1250ng/ml |
| TUDCA | y=0.1258*x+5.1*10^-5^ | 0.999 | 4.88-1250ng/ml |
| THCA | y=0.2258*x-8.379*10^-4^ | 0.999 | 4.44-1250ng/ml |
| TCDCA | y=0.2152*x+5.46*10^-4^ | 0.999 | 4.88-1250ng/ml |
| TDCA | y=0.1163*x+8.66*10^-5^ | 0.987 | 19.52-5000ng/ml |
| TCA | y=0.0756*x+2.5897*10^-6^ | 0.998 | 4.88-625ug/ml |

**3. The precision, accuracy, rate of recovery, rate of recovery, matrix effect and stability.**

The results showed that the RSD of precision ,accuracy, recovery, matrix effect and stability were less than 15%, and the mean value of them also in the range of 80%-120%, indicating that the method meets the requirements of biological sample quantitative detection methodology.

Tab.2 **Precision ,accuracy, recovery, matrix effect and stability** of the 19 BAs(n=6)

| **compound name** | **mass concentrates**  **of QC** | **Accuracy%** | **Precision RSD%** | | **rate of recovery%** | | **Matrix effect%** | | **stability%** | |
| --- | --- | --- | --- | --- | --- | --- | --- | --- | --- | --- |
|  |  |  | **Intra-**  **day** | **Within-**  **day** | **Mean value** | **RSD%** | **Mean**  **value** | **RSD%** | **Autosampler**  **72h** | **-80℃Freeze and**  **thaw for 3 times** |
| LCA | low | 101.02 | 3.6 | 4.7 | 94.46 | 6.7 | 90.45 | 5.1 | 96.53 | 108.97 |
|  | middle | 100.8 | 4.9 | 6.9 | 102.78 | 7.3 | 89.34 | 5.3 | 106.46 | 98.18 |
|  | high | 98.56 | 7.4 | 5.9 | 97.75 | 6.1 | 97.52 | 6.8 | 89.57 | 106.76 |
| DCA | low | 97.93 | 4.8 | 5.1 | 96.31 | 5.2 | 90.45 | 6 | 97.41 | 101.43 |
|  | middle | 103.41 | 6.3 | 4.8 | 106.34 | 9 | 107.12 | 3.2 | 109.22 | 106.31 |
|  | high | 95.79 | 5.4 | 5.4 | 99.43 | 7.9 | 92.43 | 5.3 | 97.12 | 108.26 |
| DHCA | low | 101.62 | 2.8 | 6.9 | 108.26 | 10.5 | 91.45 | 6.2 | 102.31 | 96.32 |
|  | middle | 99.81 | 2.7 | 4 | 89.52 | 7.9 | 102.34 | 6.1 | 98.43 | 101.94 |
|  | high | 105.76 | 5.2 | 8.9 | 93.64 | 2.1 | 109.12 | 7.8 | 97.43 | 108.27 |
| CA | low | 106.31 | 6.6 | 12.4 | 104.73 | 5.2 | 90.68 | 3.2 | 108.36 | 93.34 |
|  | middle | 99.65 | 2.3 | 3.9 | 106.44 | 1.5 | 97.89 | 7.9 | 90.87 | 98.9 |
|  | high | 93.57 | 5.6 | 5.4 | 112.38 | 6.2 | 109.56 | 6.9 | 98.17 | 105.62 |
| B-MCA | low | 97.43 | 8.9 | 9 | 97.26 | 5.3 | 109.45 | 10.9 | 96.23 | 97.47 |
|  | middle | 106.39 | 7.6 | 5.9 | 102.84 | 2.1 | 91.12 | 1.9 | 111.24 | 107.45 |
|  | high | 110.35 | 6.7 | 4.3 | 104.53 | 5.1 | 99.56 | 6.9 | 101.68 | 97.41 |
| UDCA | low | 102.74 | 10.4 | 11.2 | 97.87 | 6.9 | 101.79 | 4.1 | 113.23 | 102.22 |
|  | middle | 99.67 | 10.8 | 4.6 | 97.67 | 6 | 89.76 | 10.6 | 92.34 | 107.15 |
|  | high | 97.97 | 6.9 | 2.7 | 109.52 | 10.5 | 90.28 | 6.2 | 114.12 | 104.32 |
| HCA | low | 111.38 | 10.7 | 6.1 | 102.39 | 12.1 | 101.27 | 6.4 | 103.34 | 100.97 |
|  | middle | 100.46 | 11.3 | 8.5 | 97.95 | 10.2 | 109.54 | 9.7 | 96.32 | 99.12 |
|  | high | 93.53 | 7.7 | 2.2 | 100.66 | 4.9 | 103.58 | 8.5 | 109.25 | 103.42 |
| CDCA | low | 106.43 | 6.4 | 7.3 | 105.44 | 1.7 | 91.45 | 4.8 | 110.16 | 104.91 |
|  | middle | 97.62 | 10.3 | 6.3 | 98.6 | 8.9 | 99.02 | 10.2 | 90.78 | 97.32 |
|  | high | 98.9 | 6.8 | 5.1 | 90.67 | 6.3 | 96.78 | 10.4 | 94.22 | 108.61 |
| GUDCA | low | 102.36 | 14.2 | 7.4 | 94.32 | 7.5 | 105.76 | 1.9 | 109.21 | 101.34 |
|  | middle | 97.64 | 10.3 | 6.1 | 97.57 | 8.4 | 90.54 | 6.4 | 97.21 | 97.87 |
|  | high | 93.58 | 9.7 | 2.1 | 111.23 | 8.2 | 96.54 | 6.3 | 99.53 | 98.67 |
| GHCA | low | 95.67 | 10.8 | 3.1 | 96.56 | 6.1 | 104.67 | 12.4 | 89.18 | 89.31 |
|  | middle | 99.9 | 6.7 | 3.1 | 90.56 | 3.2 | 110.43 | 5.6 | 107.81 | 108.12 |
|  | high | 95.89 | 5.9 | 2.1 | 106.42 | 4.2 | 107.56 | 7.2 | 98.39 | 103.42 |
| GDCA | low | 108.38 | 7.9 | 7.4 | 108.82 | 6.7 | 100.67 | 9.9 | 97.43 | 106.76 |
|  | middle | 95.47 | 10.5 | 6.4 | 97.35 | 7.1 | 89.05 | 6.8 | 98.21 | 89.33 |
|  | high | 97.49 | 4.3 | 1.9 | 89.79 | 4.2 | 90.78 | 5.9 | 89.93 | 107.17 |
| GCDCA | low | 93.77 | 8.8 | 9.4 | 99.02 | 7.4 | 98.58 | 6.9 | 106.29 | 93.56 |
|  | middle | 99.93 | 6.4 | 2.1 | 88.58 | 7.5 | 91.52 | 7 | 93.46 | 101.6 |
|  | high | 101.34 | 7.1 | 4.8 | 105.52 | 3.4 | 112.56 | 3.7 | 105.24 | 90.95 |
| GCA | low | 95.38 | 8 | 5.3 | 87.27 | 7.1 | 90.89 | 7.8 | 110.45 | 90.18 |
|  | middle | 103.17 | 7.3 | 5.9 | 95.65 | 8.9 | 108.54 | 8.2 | 102.38 | 101.9 |
|  | high | 106.56 | 6.2 | 3.5 | 96.57 | 7.1 | 101.59 | 2.6 | 108.39 | 106.32 |
| TLCA | low | 101.39 | 9.8 | 6.9 | 110.13 | 2.5 | 111.48 | 9 | 96.36 | 92.87 |
|  | middle | 108.75 | 7.5 | 2.5 | 95.65 | 9.1 | 99.89 | 9.1 | 99.32 | 96.89 |
|  | high | 110.21 | 5.4 | 1.3 | 107.02 | 7.1 | 90.89 | 7.2 | 107.15 | 104.21 |
| TUDCA | low | 95.89 | 9 | 4.9 | 105.34 | 6.9 | 103.51 | 9.9 | 101.39 | 108.25 |
|  | middle | 99.46 | 8.8 | 5.3 | 98.96 | 9 | 105.78 | 6.6 | 96.87 | 110.26 |
|  | high | 109.31 | 4.3 | 5.9 | 96.68 | 6.1 | 109.91 | 7.1 | 110.42 | 104.21 |
| THCA | low | 107.49 | 12.1 | 2.1 | 107.52 | 3.2 | 105.8 | 5.9 | 102.95 | 94.61 |
|  | middle | 111.32 | 6.8 | 6.4 | 97.89 | 1.1 | 110.45 | 6.3 | 105.76 | 105.23 |
|  | high | 98.36 | 7.5 | 3.2 | 107.63 | 2.1 | 103.6 | 5.3 | 98.82 | 90.21 |
| TCDCA | low | 96.23 | 8.9 | 8.9 | 89.91 | 10.4 | 90.78 | 7.8 | 90.12 | 94.31 |
|  | middle | 106.56 | 4.8 | 5.1 | 110.32 | 4.9 | 113.61 | 7.3 | 101.24 | 110.23 |
|  | high | 90.86 | 2.6 | 4.1 | 98.44 | 7.9 | 108.67 | 8.1 | 107.23 | 113.72 |
| TDCA | low | 96.76 | 3.4 | 7.2 | 97.17 | 10.5 | 114.27 | 7.7 | 92.68 | 89.3 |
|  | middle | 97.65 | 5.5 | 5.2 | 89.54 | 6.9 | 89.9 | 6.5 | 105.23 | 100.16 |
|  | high | 103.65 | 2.4 | 3 | 106.32 | 4.5 | 94.67 | 2.3 | 112.3 | 105.27 |
| TCA | low | 100.69 | 6.9 | 8.1 | 109.81 | 2.6 | 109.53 | 6.4 | 91.97 | 92.24 |
|  | middle | 109.47 | 5.1 | 5.8 | 98.67 | 6.1 | 89.05 | 7.7 | 96.34 | 99.37 |
|  | high | 110.18 | 7.2 | 4.1 | 96.45 | 7.9 | 104.67 | 5.4 | 108.9 | 106.13 |
